# An explainable and leakage-aware machine learning model for early risk stratification of radioiodine-refractory differentiated thyroid cancer: development and single-center geographic external validation

**DOI:** 10.3389/fendo.2026.1952443

**Published:** 2026-10-02

**Authors:** Gongxun Tan, Yue Zhang, Zhenwei Mao, Chuan Zhang, Jingjing Fu, Feng Wang, Canhong Zhu, Jin Zhou, Bingquan Jin, Ying Xu, Rong Qin, Xun Shao, Lele Zhang, Shaohua Li, Qian Gu

**Affiliations:** 1 Department of Geriatrics, Affiliated People’s Hospital of Jiangsu University, Zhenjiang, Jiangsu, China; 2 Department of Nuclear Medicine, Shuyang Hospital of Chinese Traditional Medicine, Shuyang, Jiangsu, China; 3 Department of Laboratory, Affiliated Fourth People’s Hospital of Jiangsu University, Zhenjiang, Jiangsu, China; 4 Department of Nuclear Medicine, Nanjing First Hospital, Nanjing Medical University, Nanjing, Jiangsu, China; 5 Nanjing D.A. Medical Laboratory, Nanjing, Jiangsu, China; 6 Department of Oncology, Affiliated People’s Hospital of Jiangsu University, Zhenjiang, Jiangsu, China; 7 Nanjing Tianyinshan Hospital, The Affiliated Hospital of China Pharmaceutical University, Nanjing, Jiangsu, China

**Keywords:** differentiated thyroid cancer, external validation, machine learning, radioiodine refractory, random forest, risk stratification, SHAP

## Abstract

**Background:**

Radioiodine-refractory differentiated thyroid cancer (RAIR-DTC) poses a major therapeutic challenge, with loss of radioiodine avidity, limited treatment options, and worse outcomes. Current risk stratification systems were not designed to predict RAIR transformation before clinical refractoriness.

**Methods:**

This retrospective two-center study included a development cohort of 302 consecutive patients and an external validation cohort of 122. Candidate predictors were restricted to baseline demographic, clinicopathological, molecular, and biochemical variables available at or before first postoperative RAI administration; all post-treatment, response-related, and time-dependent variables were excluded *a priori*. Six machine-learning algorithms were developed with repeated cross-validation and compared by discrimination, probability accuracy, and clinical utility. Model interpretation used SHapley Additive exPlanations (SHAP). External transportability was assessed by discrimination, calibration, decision-curve analysis, and validation of prespecified risk groups.

**Results:**

Forty-six patients (15.2%) developed RAIR-DTC in the development cohort and 18 (14.8%) in the external cohort. Random forest was selected for the most favorable balance of cross-validated discrimination and probability accuracy, despite comparable external discrimination from XGBoost and the stacked ensemble. It achieved an area under the receiver operating characteristic curve (AUC) of 0.896 and Brier score of 0.078 in the internal test cohort, with robust external discrimination (AUC, 0.892; PR-AUC, 0.597; sensitivity, 0.778; specificity, 0.798). Raw external calibration was poor (calibration slope 0.304; observed-to-expected ratio 0.613), indicating that ranking was preserved whereas absolute risk estimates required local recalibration. SHAP analysis identified age ≥55 years, TERT promoter (TERTp) mutation, vascular invasion, initial stimulated thyroglobulin (sTg), and extrathyroidal extension (ETE) as the dominant contributors. Training-derived quantile cut points generated three-tier risk groups, identifying a low-risk subgroup with zero events in the external cohort and a low event rate (3.3%) in the internal test set, while enriching the high-risk subgroup to event rates of 75.0% and 37.2%, respectively, with 95% confidence intervals of 47.6%-92.7% and 23.0%-53.3%, respectively.

**Conclusions:**

An explainable, leakage-aware random forest based exclusively on routinely available baseline variables provided robust external discrimination and clinically meaningful early risk stratification for RAIR-DTC. Preserved discrimination did not guarantee transportable probability estimates, underscoring that external calibration and site-specific recalibration are essential prerequisites for responsible clinical deployment.

## 1 Introduction

Differentiated thyroid cancer (DTC) accounts for the majority of thyroid malignancies and generally confers an excellent prognosis after surgery, risk-adapted radioactive iodine (RAI) therapy, and thyroid-stimulating hormone suppression (1, 2). However, a clinically important subset of patients develop radioiodine-refractory disease (RAIR-DTC), defined by loss or marked reduction of iodine avidity, disease progression despite adequate RAI exposure, or heterogeneous uptake across lesions (3, 4). Approximately two-thirds of patients with distant metastases eventually lose iodine avidity, and 10-year survival in this group falls to about 10%, compared with 56% among those whose metastases remain RAI-avid (5). Once refractoriness develops, disease control becomes more challenging, and management may shift to local ablative therapies, multikinase inhibitors, selective targeted agents, or redifferentiation strategies (6, 7).

Current risk assessment is based on clinicopathological staging and response-to-therapy systems, such as the American Joint Committee on Cancer (AJCC) TNM, the American Thyroid Association (ATA) risk stratification, and dynamic response criteria (2, 9, 10). Although these frameworks are indispensable for prognosis and follow-up planning, they were not specifically designed to estimate the individualized probability of RAIR-DTC before refractoriness becomes clinically evident. Molecular alterations, particularly TERT promoter (TERTp) mutations and their interaction with BRAF-driven signaling, promote dedifferentiation by downregulating sodium iodide symporter (NIS) expression and impairing iodine uptake (10, 11). Initial stimulated thyroglobulin (sTg), vascular invasion, extrathyroidal extension (ETE), and distant metastatic (DM) status may also capture tumor burden and biologic aggressiveness (12). Their joint predictive value is likely nonlinear and may not be fully captured by a single conventional regression model. Therefore, a clinically useful model should identify high-risk patients before irreversible loss of iodine avidity, rather than after RAIR-DTC has already developed. Patients identified as high risk could then be prioritized for intensified structural and biochemical surveillance, more critical appraisal of repeated RAI courses, and earlier referral for systemic or redifferentiation therapy, whereas low-risk patients could be spared unnecessary intensification (6–8).

Clinical translation, however, requires more than high discrimination. Models must be developed using information available at the intended prediction time and validated across institutions with assessment of calibration and transportability. Machine learning methods can model nonlinear relations and higher-order interactions, but high apparent accuracy may be misleading when post-treatment variables are inadvertently included or when performance is reported only in the derivation sample (14). In RAIR-DTC specifically, previous work has largely reported single-center associations between individual clinicopathological or molecular features and refractoriness, without multivariable model development, external validation, or explicit control of post-treatment information (13). Here, leakage denotes the use of predictors that become observable only after the intended prediction time, principally post-RAI treatment response, follow-up imaging findings, and time-dependent thyroglobulin trends, which inflate apparent accuracy but are unavailable at the moment the prediction would be made. We therefore designed a leakage-aware, externally validated comparison of six algorithms using only prespecified baseline variables. The objectives were to: (1) compare six algorithms and select a candidate model for external evaluation; (2) quantify discrimination and calibration in an independent center; (3) explain the model using SHAP; and (4) develop clinically interpretable risk groups and probability-based operating thresholds.

## 2 Materials and methods

### 2.1 Study design and participants

This two-center study included a development cohort of 302 patients treated at Nanjing First Hospital, Nanjing Medical University and an external validation cohort of 122 patients from the Affiliated People’s Hospital of Jiangsu University. In the development cohort, 46 patients developed RAIR-DTC; the external cohort contained 18 events. Eligible patients had histologically confirmed DTC, underwent total or near-total thyroidectomy and postoperative RAI management, and had baseline clinicopathological, molecular, and biochemical variables available for model development. Variable-specific missingness was <2% and was handled using prespecified imputation procedures. Patients with incomplete molecular testing, insufficient follow-up (defined as <6 months after the date of first postoperative RAI administration), prior history of other malignancies, or aggressive histological variants (e.g., tall cell, columnar cell, hobnail, or anaplastic components) were excluded. The index date for prediction was the date of the first postoperative RAI administration. The primary prediction target was binary and was defined as the diagnosis of RAIR-DTC at any time during the available follow-up after the index date; the model was not designed to estimate RAIR-DTC risk within a prespecified fixed time horizon. The patient selection process is detailed in a CONSORT-style flow diagram (**Figure 1**). The development cohort was split by outcome-stratified random sampling (7:3) into a training set (n=212; 32 events) and an internal test set (n=90; 14 events). The external cohort was not used for feature selection, hyperparameter tuning, threshold selection, or model fitting. This study is based on the same institutional patient cohort as our companion article (15), which addressed distinct descriptive questions; the present work develops and validates a prediction model.

**Figure 1:**
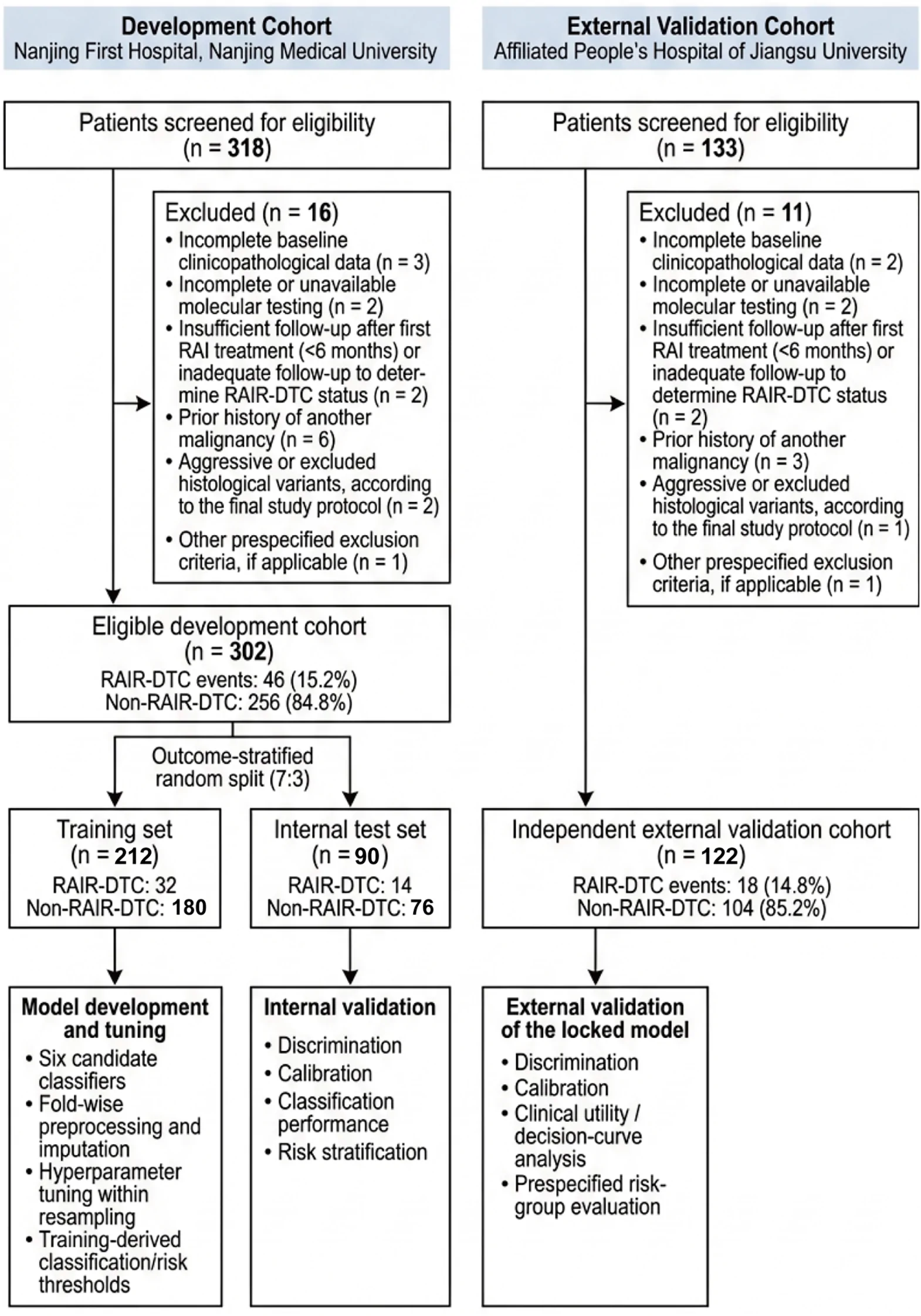
Study flow diagram of patient selection. The diagram shows the number of patients initially screened at each center, reasons for exclusion, and the final number of patients in the development cohort (Nanjing First Hospital, n=302) and external validation cohort (Affiliated People’s Hospital of Jiangsu University, n=122), as well as the approximate 7:3 split of the development cohort into training (n=212) and internal test (n=90) sets.

RAIR-DTC was defined according to the 2025 Chinese management guidelines (3) and the 2025 ATA guidelines (4), requiring at least one of: (1) absence of radioiodine uptake in known metastatic or structurally persistent disease from the first post-therapeutic ¹³¹I evaluation; (2) loss of previously documented radioiodine avidity in one or more lesions during follow-up; (3) spatial heterogeneity of iodine avidity, with radioiodine uptake retained in some lesions but absent in other structurally evident lesions; (4) persistent radioiodine avidity with disease progression within 1 year after RAI therapy, manifested by gradual enlargement of existing lesions, the appearance of new lesions, or a continuous increase in serum Tg or anti-thyroglobulin antibody (TgAb) levels; or (5) persistent/progressive disease after a cumulative RAI activity ≥600 mCi (22.2 GBq). The distribution of patients meeting each individual RAIR-DTC criterion and detailed description of diagnostic basis are provided in **Supplementary Tables S1**, **S2**. The criteria were not mutually exclusive, and classification was based on the complete longitudinal clinical, biochemical, and imaging record rather than on sTg elevation alone. RAIR-DTC classification was independently reviewed by two experienced nuclear medicine physicians, with disagreements resolved by consensus. The first post-treatment assessment was performed 3–6 months after the first postoperative I-131 administration, irrespective of the number of subsequent RAI courses. Thereafter, patients initially classified as low-risk were followed every 6–12 months, whereas those at intermediate-to-high recurrence risk were followed every 3–6 months. RAIR-DTC status was ascertained throughout this standardized longitudinal follow-up, rather than at a single fixed time point.

### 2.2 Candidate predictors and leakage control

Candidate predictors comprised sex, age ≥55 years, histologic subtype (papillary vs. follicular), tumor size (modelled as a continuous variable based on maximum diameter), multifocality, ETE, vascular invasion, lymph node metastasis (LNM), DM, *BRAF* V600E mutation, TERTp mutation, and initial pre-RAI sTg (modelled as a continuous variable). All candidate predictors were required to be available at or before the index date. No information generated after RAI administration was used as a predictor. The exact measurement time windows for each predictor are provided in **Supplementary Table S3**. Variables available only after treatment response or after the RAIR-DTC outcome—including follow-up duration, recurrence recorded after the index assessment, post-treatment WBS findings, ¹^8^F-FDG PET/CT results, sTg at the time of RAIR-DTC, targeted therapy, drug resistance, and survival status—were excluded *a priori* from model training. Cumulative ¹³¹I activity and number of RAI treatments were also excluded because their valid use requires a prespecified landmark time, which would introduce time-dependent bias. All predictors were collected at or before the index date. The exact coding of each variable used in the machine learning models is provided in **Supplementary Table S4**. **Table 1** includes some of these excluded variables (e.g., recurrence, cumulative I-131 ≥600 mCi, death during follow-up) for descriptive purposes only; they were not used in any model development or validation.

**Table 1:** Baseline characteristics of the training, internal test, and external validation cohorts.

Variable	Category	Training (n=212)	Internal test (n=90)	External (n=122)	P value (overall across cohorts)	SMD (internal vs external)
RAIR-DTC event	Yes	32 (15.1%)	14 (15.6%)	18 (14.8%)	0.987	0.022
	No	180 (84.9%)	76 (84.4%)	104 (85.2%)		
Age >= 55 years	Yes	56 (26.4%)	19 (21.1%)	42 (34.4%)	0.087	0.301
	No	156 (73.6%)	71 (78.9%)	80 (65.6%)		
sTg >10.77 ng/mL†	Yes	82 (38.7%)	36 (40.0%)	52 (42.6%)	0.778	0.053
	No	130 (61.3%)	54 (60.0%)	70 (57.4%)		
Tumor size >= 2 cm ‡	Yes	79 (37.3%)	28 (31.1%)	44 (36.1%)	0.589	0.105
	No	133 (62.7%)	62 (68.9%)	78 (63.9%)		
Distant metastasis	Yes	20 (9.4%)	9 (10.0%)	17 (13.9%)	0.426	0.121
	No	192 (90.6%)	81 (90.0%)	105 (86.1%)		
Cumulative 131I >= 600 mCi (descriptive only)	Yes	3 (1.4%)	0 (0.0%)	1 (0.8%)	0.501	0.129
	No	209 (98.6%)	90 (100.0%)	121 (99.2%)		
Recurrence (descriptive only)	Yes	40 (18.9%)	17 (18.9%)	26 (21.3%)	0.849	0.06
	No	172 (81.1%)	73 (81.1%)	96 (78.7%)		
ETE positive	Yes	72 (34.0%)	32 (35.6%)	46 (37.7%)	0.788	0.045
	No	140 (66.0%)	58 (64.4%)	76 (62.3%)		
Vascular invasion	Yes	38 (17.9%)	13 (14.4%)	17 (13.9%)	0.568	0.015
	No	174 (82.1%)	77 (85.6%)	105 (86.1%)		
LNM positive	Yes	166 (78.3%)	72 (80.0%)	89 (73.0%)	0.408	0.167
	No	46 (21.7%)	18 (20.0%)	33 (27.0%)		
Multifocality	Yes	91 (42.9%)	48 (53.3%)	76 (62.3%)	0.003	0.182
	No	121 (57.1%)	42 (46.7%)	46 (37.7%)		
*BRAF* V600E mutation	Yes	164 (77.4%)	66 (73.3%)	110 (90.2%)	0.003	0.446
	No	48 (22.6%)	24 (26.7%)	12 (9.8%)		
TERTp mutation	Yes	16 (7.5%)	2 (2.2%)	29 (23.8%)	<0.001	0.676
	No	196 (92.5%)	88 (97.8%)	93 (76.2%)		
Death during follow-up (descriptive only)	Yes	7 (3.3%)	1 (1.1%)	6 (4.9%)	0.309	0.224
	No	205 (96.7%)	89 (98.9%)	116 (95.1%)		

SMD, standardized mean difference. SMD > 0.2 indicates a potentially meaningful difference. † sTg was modelled as a continuous variable in the machine learning models; the 10.77 ng/mL cutoff is presented only for descriptive purposes. ‡ Tumor size was modelled as a continuous variable in the machine learning models; the 2 cm cutoff is presented only for descriptive purposes. Age ≥55 years was prespecified because 55 years is the AJCC 8th-edition age threshold used in DTC staging (9). The 10.77 ng/mL cutoff was derived in our published companion article (15). P values compare all three cohorts.

### 2.3 Model development and tuning

Given the limited number of outcome events, we avoided deep learning approaches and restricted model complexity through repeated cross-validation and external validation. Six classifiers were evaluated: standard logistic regression, LASSO logistic regression, random forest, radial-basis support vector machine (SVM), extreme gradient boosting (XGBoost), and a stacked ensemble combining predictions from the five base learners via a logistic meta-learner. Hyperparameter tuning was nested within cross-validation: an inner 5-fold loop was used for tuning and an outer 5-fold loop for out-of-fold (OOF) performance estimation, repeated 100 times. Hyperparameters were optimized over prespecified ranges (**Supplementary Table S5**), with the random seed set to 2024. Final model selection used only OOF performance from the training set. Random forest was selected for the best overall balance of discrimination and probability accuracy: its OOF ROC AUC was 0.944, essentially the same as XGBoost (0.945), while its OOF Brier score was the lowest (0.059; **Table 2**). The external validation cohort was sequestered and not accessed until the final model was locked. The final random-forest tuning parameters were mtry = 6 and min.node.size = 3; the LASSO penalty was λ = 0.01374 (approximately 0.014). The random forest model used 1000 trees, equal class weights c(0.5, 0.5), and sampling with replacement. For XGBoost, early stopping with a patience of 50 rounds yielded an optimal boosting iteration of 507 rounds (**Supplementary Table S5**). The complete preprocessing pipeline, including scaling for SVM and one-hot encoding for categorical variables, was nested within each cross-validation training fold. Missing data (overall <2%) were handled by median imputation for continuous variables and mode imputation for categorical variables, performed within cross-validation folds to prevent leakage (**Supplementary Table S6**). This study was reported according to the TRIPOD guidelines for prediction model development and validation.

**Table 2:** Out-of-fold performance of candidate models in the training set (n=212; 32 events).

Model	OOF ROC AUC (95% CI)	OOF PR-AUC (95% CI)	OOF Brier score (95% CI)
Random forest	0.944 (0.894-0.976)	0.815 (0.693-0.898)	0.059 (0.042-0.077)
XGBoost	0.945 (0.910-0.973)	0.806 (0.686-0.893)	0.061 (0.042-0.083)
SVM	0.930 (0.876-0.967)	0.758 (0.616-0.863)	0.066 (0.046-0.089)
Stacked ensemble	0.929 (0.870-0.969)	0.730 (0.571-0.863)	0.070 (0.050-0.090)
LASSO	0.908 (0.851-0.952)	0.639 (0.481-0.811)	0.075 (0.055-0.096)
Logistic	0.860 (0.775-0.928)	0.650 (0.483-0.792)	0.077 (0.054-0.102)

### 2.4 Model evaluation

Discrimination was quantified using ROC AUC and PR-AUC. Accuracy, sensitivity, specificity, positive predictive value (PPV), negative predictive value (NPV), and F1 score were calculated at model-specific thresholds selected in the training data by the Youden index and then carried forward unchanged (**Supplementary Table S7**). 95% confidence intervals for all performance metrics were estimated using 2000 bootstrap resamples. Probability accuracy was assessed using the Brier score. Calibration was evaluated using calibration intercept, calibration slope, observed-to-expected (O/E) ratio, graphical calibration curves, and integrated calibration index (ICI) where estimable. Clinical utility was explored using decision-curve analysis (net benefit across threshold probabilities). Pairwise AUC comparisons used DeLong tests with Holm adjustment for multiple comparisons. Confidence intervals were estimated methods where reported.

Calibration results for the random forest were calculated from the raw predicted probabilities. The raw-probability calibration statistics are reported in **Supplementary Table S12**.

### 2.5 Explainability and risk stratification

For the selected random forest model, global feature importance was calculated from mean absolute SHAP values. SHAP values were generated from the final model fitted on the entire development cohort after internal validation. SHAP beeswarm and dependence plots were used to display the direction, magnitude, and potential nonlinearity of feature effects. Three risk categories were defined using the 33rd and 67th percentiles of the predicted-risk distribution in the training set (n=212). These cut points (0.000 and 0.003, approximately) were selected *a priori* using a purely quantile-based, outcome-agnostic method and were not influenced by observed outcomes. These fixed cut points were applied to the internal and external validation cohorts without modification. The three-tier risk stratification is presented as an exploratory analysis to demonstrate the model’s ability to separate risk, rather than as a validated clinical decision rule.

### 2.6 Statistical analysis

Categorical variables are reported as counts and percentages, and continuous variables as median with interquartile range. Standardized mean differences (SMD) were calculated to quantify case-mix differences between the internal and external cohorts. Two-sided P values < 0.05 were considered statistically significant. Analyses were conducted in R version 4.3.1, using packages caret (6.0-94), randomForest (4.7-1.1), xgboost (1.7.6), kernlab (0.9-32), glmnet (4.1-8), pROC (1.18.5), and shapviz (0.9.3). Differences between AUCs were compared using the DeLong test for correlated ROC curves. The Holm method was applied to adjust P values for multiple pairwise comparisons.

### 2.7 Ethics

The study was performed in accordance with the Declaration of Helsinki. Ethics approval was obtained from the Ethics Committee of Nanjing First Hospital, Nanjing Medical University (approval no. 2024-NY-078). Informed consent was waived due to the retrospective nature, except for the publication of any potentially identifiable data, for which consent was obtained.

## 3 Results

### 3.1 Cohort characteristics

The development cohort included 302 patients, of whom 46 (15.2%) developed RAIR-DTC. The external cohort included 122 patients, of whom 18 (14.8%) developed RAIR-DTC. In the development cohort, median follow-up was 39.5 months (IQR: 7.0-132.0) for patients who developed RAIR-DTC and 33.0 months (IQR: 1.0-172.0) for those who did not. In the external cohort, median follow-up was 16.0 months (IQR: 1.0-115.0) for RAIR-DTC patients and 16.0 months (IQR: 1.0-158.0) for non-RAIR-DTC patients. To further address the time-dependent nature of RAIR-DTC, we performed an exploratory Cox proportional hazards analysis using a reduced set of eight baseline predictors available at the index date: age ≥55 years, TERTp mutation, vascular invasion, initial sTg, ETE, *BRAF* V600E mutation, LNM, and multifocality. Given the limited number of RAIR-DTC events, the exploratory survival analysis used a reduced predictor set to limit model complexity and was not intended to reproduce the full machine-learning predictor set. In the development cohort, age ≥55 years (HR, 3.21; 95% CI, 1.89-5.45; P < 0.001), TERTp mutation (HR, 2.98; 95% CI, 1.76-5.04; P < 0.001), vascular invasion (HR, 2.12; 95% CI, 1.28-3.51; P = 0.003), and sTg (per 10-unit increase, HR, 1.08; 95% CI, 1.02-1.14; P = 0.012) were significantly associated with a shorter time to RAIR-DTC development (**Supplementary Table S8**). These exploratory findings indicate that several predictors contributing to binary RAIR-DTC classification were also associated with time to RAIR-DTC; however, this analysis does not convert the primary model into a fixed-horizon survival prediction model.

Event prevalence was similar across the training set (15.1%), internal test set (15.6%), and external cohort (14.8%). The training and internal test sets were generally well balanced. Compared with the internal test set, the external cohort had higher frequencies of *BRAF* V600E mutation (90.2% vs. 73.3%) and TERTp mutation (23.8% vs. 2.2%), highlighting clinically relevant case-mix differences that provide a stringent test of transportability (**Table 1**). Standardized mean differences (SMD) confirmed meaningful case-mix shifts for age ≥55 years (SMD = 0.301), death during follow-up (SMD = 0.224), *BRAF* V600E mutation (SMD = 0.446), and TERTp mutation (SMD = 0.676), while other variables showed smaller differences (SMD <0.2) (**Table 1**; **Supplementary Table S10**). These case-mix differences, particularly in molecular features, may have contributed to the observed calibration drift between cohorts.

The distribution of RAIR-DTC criteria in the development cohort (n=46): criterion 1, 27 patients (58.7%); criterion 2, 1 patient (2.2%); criterion 3, 6 patients (13.0%); criterion 4, 16 patients (34.8%); and criterion 5, 2 patients (4.3%). In the external cohort (n=18), the corresponding numbers were 10 (55.6%), 0 (0.0%), 2 (11.1%), 6 (33.3%), and 1 (5.6%), respectively. Criteria were not mutually exclusive (**Supplementary Table S9**), so counts can overlap. Multiple criteria were met by 6 patients (13.0%) in the development cohort and 1 patient (5.6%) in the external cohort. The composite endpoint therefore represents clinically heterogeneous manifestations of radioiodine refractoriness.

### 3.2 Cross-validated model selection

In the out-of-fold model-development analysis, XGBoost achieved the highest ROC AUC (0.945), followed closely by random forest (0.944) and SVM (0.930). Random forest had the lowest OOF Brier score (0.059), indicating the best probability accuracy among the six candidates in this analysis. Logistic regression, LASSO, and the stacked ensemble had OOF AUCs of 0.860, 0.908, and 0.929, respectively (**Table 2**).

### 3.3 Internal and external validation

In the internal test set, model discrimination ranged from good to excellent, with AUCs from 0.850 to 0.901. XGBoost showed the highest AUC of 0.901 (95% CI, 0.778-1.000), followed closely by logistic regression (0.900; 95% CI, 0.777-1.000) and random forest (0.896; 95% CI, 0.781-1.000). The stacked ensemble yielded an AUC of 0.883 (95% CI, 0.746-1.000), LASSO 0.891 (95% CI, 0.764-1.000), and SVM 0.850 (95% CI, 0.721-0.979). At the Youden-derived cutoffs, sensitivities ranged from 0.643 (random forest) to 0.857 (stacked ensemble), specificities from 0.947 to 0.974, and Brier scores from 0.069 to 0.080 (**Table 3**; **Figure 2A**).

**Table 3:** Performance of all candidate models in the internal test, external validation, and training cohorts.

Cohort	Model	n	Events	AUC (95% CI)	PR-AUC (95% CI)	Sensitivity (95% CI)	Specificity (95% CI)	PPV (95% CI)	NPV (95% CI)	F1 (95% CI)	Brier score (95% CI)	Training-derived cut-off
Internal validation	LASSO	90	14	0.891 (0.764-1.000)	0.763 (0.508-0.946)	0.714 (0.455-0.929)	0.961 (0.912-1.000)	0.769 (0.526-1.000)	0.948 (0.893-0.988)	0.741 (0.522-0.903)	0.072 (0.032-0.118)	0.161
	Logistic	90	14	0.900 (0.777-1.000)	0.782 (0.534-0.955)	0.714 (0.455-0.929)	0.961 (0.912-1.000)	0.769 (0.526-1.000)	0.948 (0.893-0.988)	0.741 (0.522-0.903)	0.072 (0.031-0.119)	0.125
	Random forest	90	14	0.896 (0.781-1.000)	0.775 (0.523-0.956)	0.643 (0.375-0.895)	0.974 (0.934-1.000)	0.818 (0.556-1.000)	0.937 (0.875-0.987)	0.720 (0.476-0.895)	0.078 (0.036-0.128)	0.245
	SVM	90	14	0.850 (0.721-0.979)	0.709 (0.445-0.915)	0.714 (0.455-0.929)	0.961 (0.912-1.000)	0.769 (0.526-1.000)	0.948 (0.893-0.988)	0.741 (0.522-0.903)	0.080 (0.040-0.127)	0.075
	Stacked ensemble	90	14	0.883 (0.746-1.000)	0.778 (0.522-0.958)	0.857 (0.643-1.000)	0.947 (0.892-0.987)	0.750 (0.533-0.944)	0.973 (0.931-1.000)	0.800 (0.621-0.933)	0.075 (0.031-0.126)	0.038
	XGBoost	90	14	0.901 (0.778-1.000)	0.788 (0.542-0.959)	0.714 (0.455-0.929)	0.961 (0.912-1.000)	0.769 (0.526-1.000)	0.948 (0.893-0.988)	0.741 (0.522-0.903)	0.069 (0.028-0.117)	0.121
External validation	LASSO	122	18	0.880 (0.817-0.944)	0.533 (0.302-0.750)	0.778 (0.571-0.947)	0.750 (0.670-0.828)	0.350 (0.200-0.500)	0.951 (0.904-0.988)	0.483 (0.308-0.627)	0.185 (0.124-0.248)	0.161
	Logistic	122	18	0.879 (0.810-0.947)	0.565 (0.318-0.783)	0.778 (0.571-0.947)	0.769 (0.687-0.845)	0.368 (0.214-0.515)	0.952 (0.906-0.989)	0.500 (0.320-0.645)	0.191 (0.126-0.257)	0.125
	Random forest	122	18	0.892 (0.829-0.955)	0.597 (0.346-0.810)	0.778 (0.571-0.947)	0.798 (0.723-0.873)	0.400 (0.241-0.567)	0.954 (0.909-0.989)	0.528 (0.350-0.677)	0.144 (0.094-0.194)	0.245
	SVM	122	18	0.854 (0.772-0.937)	0.381 (0.232-0.660)	0.833 (0.640-1.000)	0.760 (0.677-0.838)	0.375 (0.227-0.523)	0.963 (0.919-1.000)	0.517 (0.346-0.656)	0.127 (0.086-0.170)	0.075
	Stacked ensemble	122	18	0.879 (0.808-0.951)	0.572 (0.321-0.791)	0.889 (0.720-1.000)	0.740 (0.657-0.819)	0.372 (0.227-0.515)	0.975 (0.935-1.000)	0.525 (0.360-0.667)	0.187 (0.124-0.254)	0.038
	XGBoost	122	18	0.893 (0.831-0.954)	0.591 (0.333-0.796)	0.778 (0.571-0.947)	0.769 (0.687-0.845)	0.368 (0.214-0.515)	0.952 (0.906-0.989)	0.500 (0.320-0.645)	0.173 (0.114-0.234)	0.121
Training	LASSO	212	32	0.978 (0.963-0.994)	0.889 (0.793-0.960)	1.000 (1.000-1.000)	0.878 (0.827-0.924)	0.593 (0.457-0.721)	1.000 (1.000-1.000)	0.744 (0.627-0.838)	0.043 (0.027-0.060)	0.161
	Logistic	212	32	0.980 (0.965-0.994)	0.891 (0.792-0.964)	1.000 (1.000-1.000)	0.889 (0.839-0.933)	0.615 (0.475-0.746)	1.000 (1.000-1.000)	0.762 (0.644-0.855)	0.042 (0.025-0.062)	0.125
	Random forest	212	32	0.978 (0.961-0.994)	0.897 (0.802-0.963)	0.906 (0.794-1.000)	0.950 (0.915-0.983)	0.763 (0.619-0.902)	0.983 (0.960-1.000)	0.829 (0.722-0.918)	0.043 (0.026-0.062)	0.245
	SVM	212	32	0.982 (0.968-0.996)	0.909 (0.822-0.970)	1.000 (1.000-1.000)	0.883 (0.832-0.929)	0.604 (0.467-0.736)	1.000 (1.000-1.000)	0.753 (0.636-0.848)	0.047 (0.031-0.066)	0.075
	Stacked Ensemble	212	32	0.961 (0.933-0.990)	0.839 (0.705-0.936)	0.969 (0.897-1.000)	0.856 (0.800-0.905)	0.544 (0.410-0.677)	0.994 (0.980-1.000)	0.697 (0.571-0.800)	0.053 (0.030-0.079)	0.038
	XGBoost	212	32	0.982 (0.969-0.996)	0.910 (0.824-0.972)	1.000 (1.000-1.000)	0.889 (0.839-0.933)	0.615 (0.475-0.746)	1.000 (1.000-1.000)	0.762 (0.644-0.855)	0.037 (0.023-0.053)	0.121

**Figure 2:**
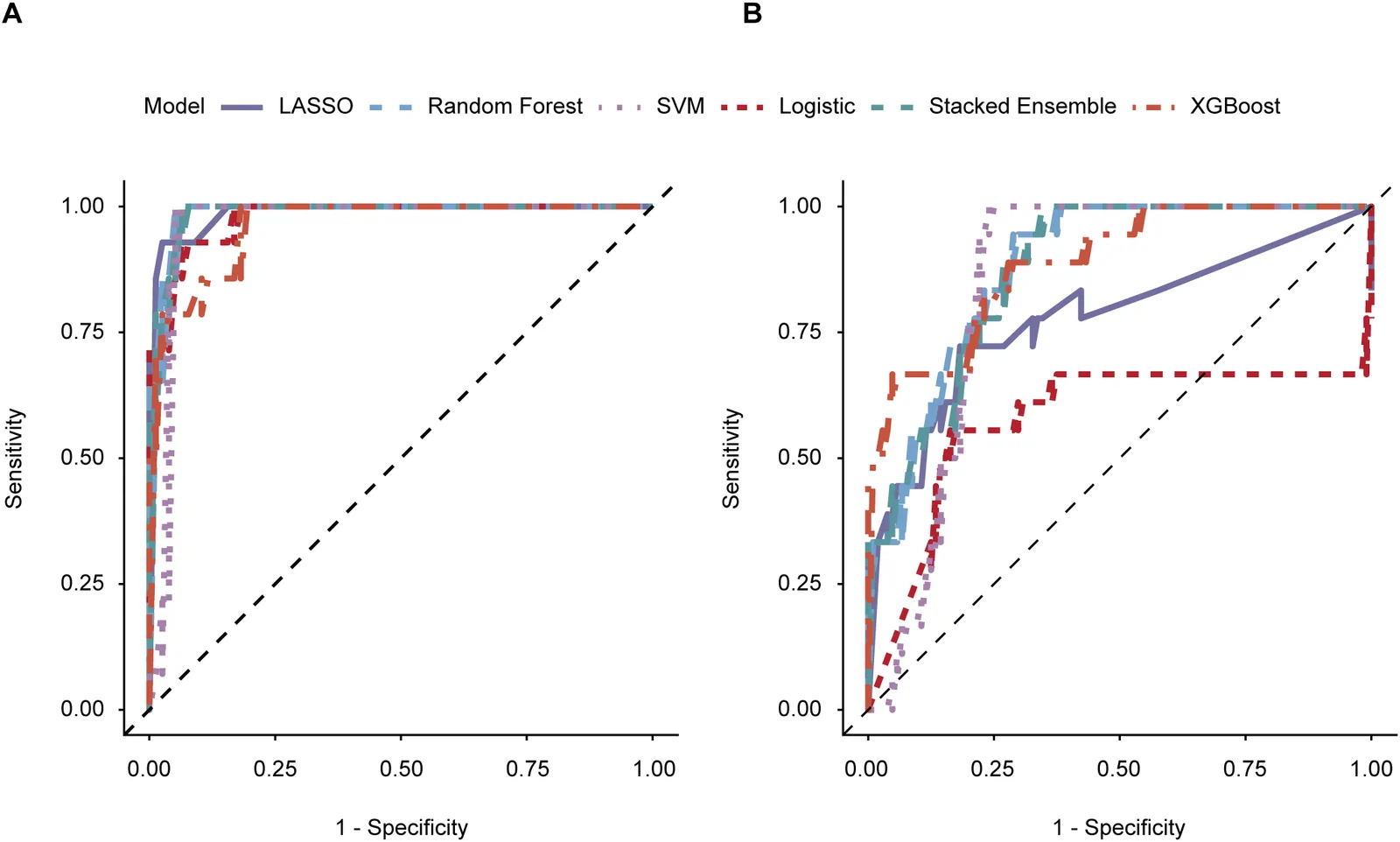
Receiver operating characteristic curves in the internal **(A)** and external **(B)** test cohort. Curves compare LASSO, random forest, support vector machine, logistic regression, stacked ensemble, and XGBoost across classification thresholds.

In the external validation cohort, all models maintained useful discrimination. XGBoost attained the highest AUC of 0.893 (95% CI, 0.831-0.954), followed by random forest (0.892; 95% CI, 0.829-0.955), LASSO (0.880; 95% CI, 0.817-0.944), logistic regression (0.879; 95% CI, 0.810-0.947), stacked ensemble (0.879; 95% CI, 0.808-0.951), and SVM (0.854; 95% CI, 0.772-0.937). Random forest showed a sensitivity of 0.778, specificity of 0.798, NPV of 0.954, and Brier score of 0.144 (**Table 3**). Pairwise DeLong tests found no statistically significant AUC differences between any model pair (all unadjusted P >0.05; **Table 4**); Holm correction did not change this conclusion. External receiver operating characteristic curves are shown in **Figure 2B**, and precision-recall curves for the internal and external cohorts in **Figures 3A, B**.

**Table 4:** Pairwise DeLong comparisons of AUCs in the external validation cohort.

Model 1	Model 2	AUC Model 1	AUC difference (model 1-model 2)	P value (unadjusted)
Logistic	RandomForest	0.879	-0.013	0.866
Logistic	SVM	0.879	0.025	0.898
Logistic	LASSO	0.879	-0.002	0.939
Logistic	XGBoost	0.879	-0.014	0.846
Logistic	Stacked Ensemble	0.879	-0.001	0.988
Random forest	SVM	0.892	0.038	0.744
Random forest	LASSO	0.892	0.012	0.869
Random forest	XGBoost	0.892	-0.001	0.988
Random forest	Stacked Ensemble	0.892	0.013	0.813
SVM	LASSO	0.854	-0.026	0.892
SVM	XGBoost	0.854	-0.038	0.714
SVM	Stacked Ensemble	0.854	-0.025	0.903
LASSO	XGBoost	0.880	-0.012	0.866
LASSO	Stacked Ensemble	0.880	0.001	0.973
XGBoost	Stacked Ensemble	0.893	0.013	0.787

AUCs were recalculated from external validation prediction probabilities. Pairwise comparisons were performed using the DeLong test; Holm adjustment did not change the conclusion.

**Figure 3:**
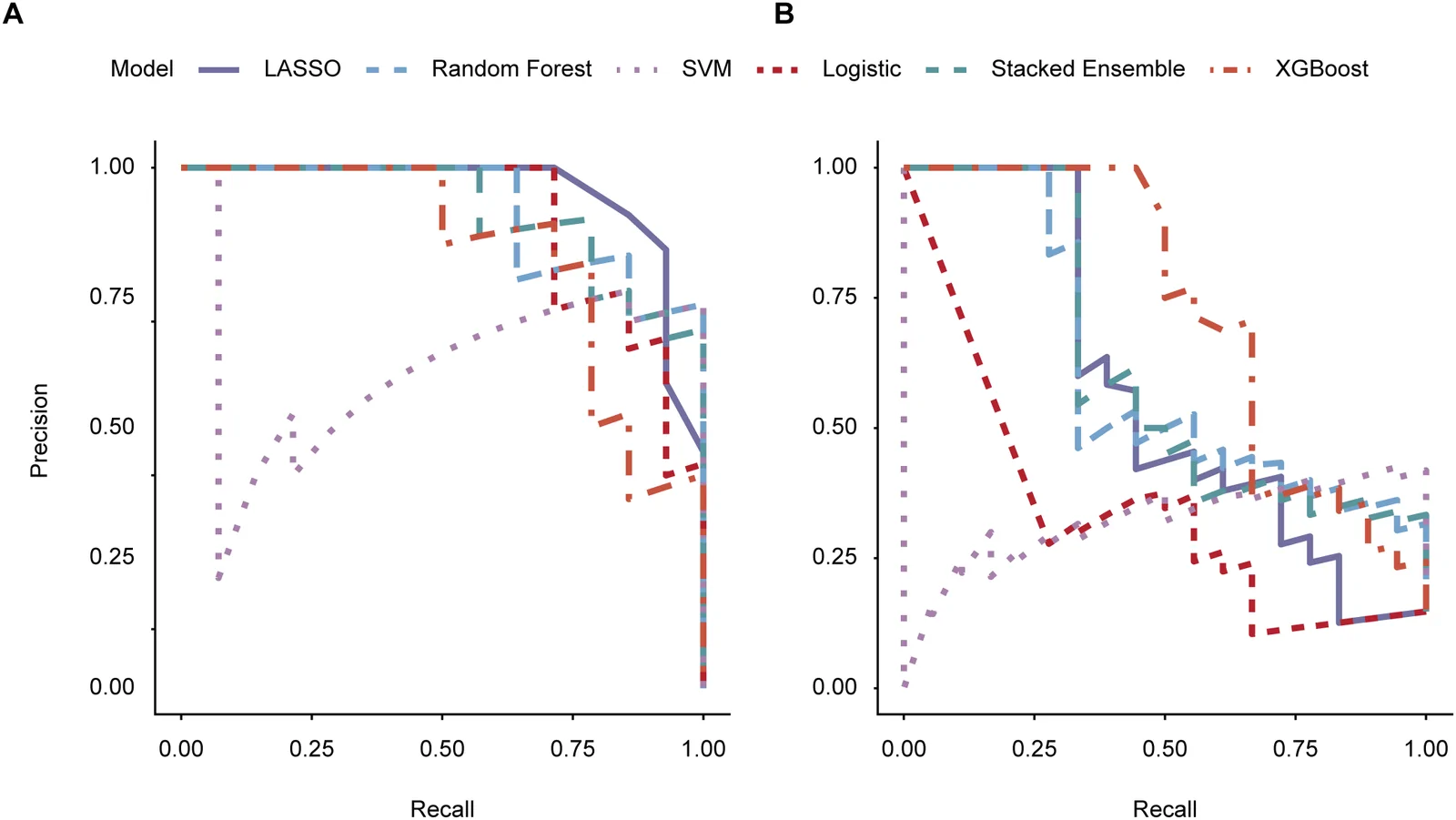
Precision-recall curves in the internal **(A)** and external **(B)** test cohort. Precision is plotted against recall for all six candidate models; this representation complements ROC analysis in the presence of a relatively low event prevalence.

### 3.4 Model explanation

The leading random forest features by mean absolute SHAP value were age ≥55 years (0.0948), TERTp mutation (0.0528), vascular invasion (0.0501), initial pre-RAI sTg (0.0250), ETE (0.0238), *BRAF* V600E (0.0219), and LNM (0.0209). The SHAP beeswarm plot showed that older age, TERTp mutation, vascular invasion, and higher initial pre-RAI sTg generally shifted predictions toward greater RAIR-DTC risk. The dependence plot for sTg suggested a nonlinear association, with risk increasing at lower to moderate concentrations and flattening or declining at very high values; however, this pattern should be interpreted cautiously because of sparse observations at the extremes (only 5 patients had sTg > 50 ng/mL in the development cohort, and 2 in the external cohort) (**Figures 4**, **5**).

**Figure 4:**
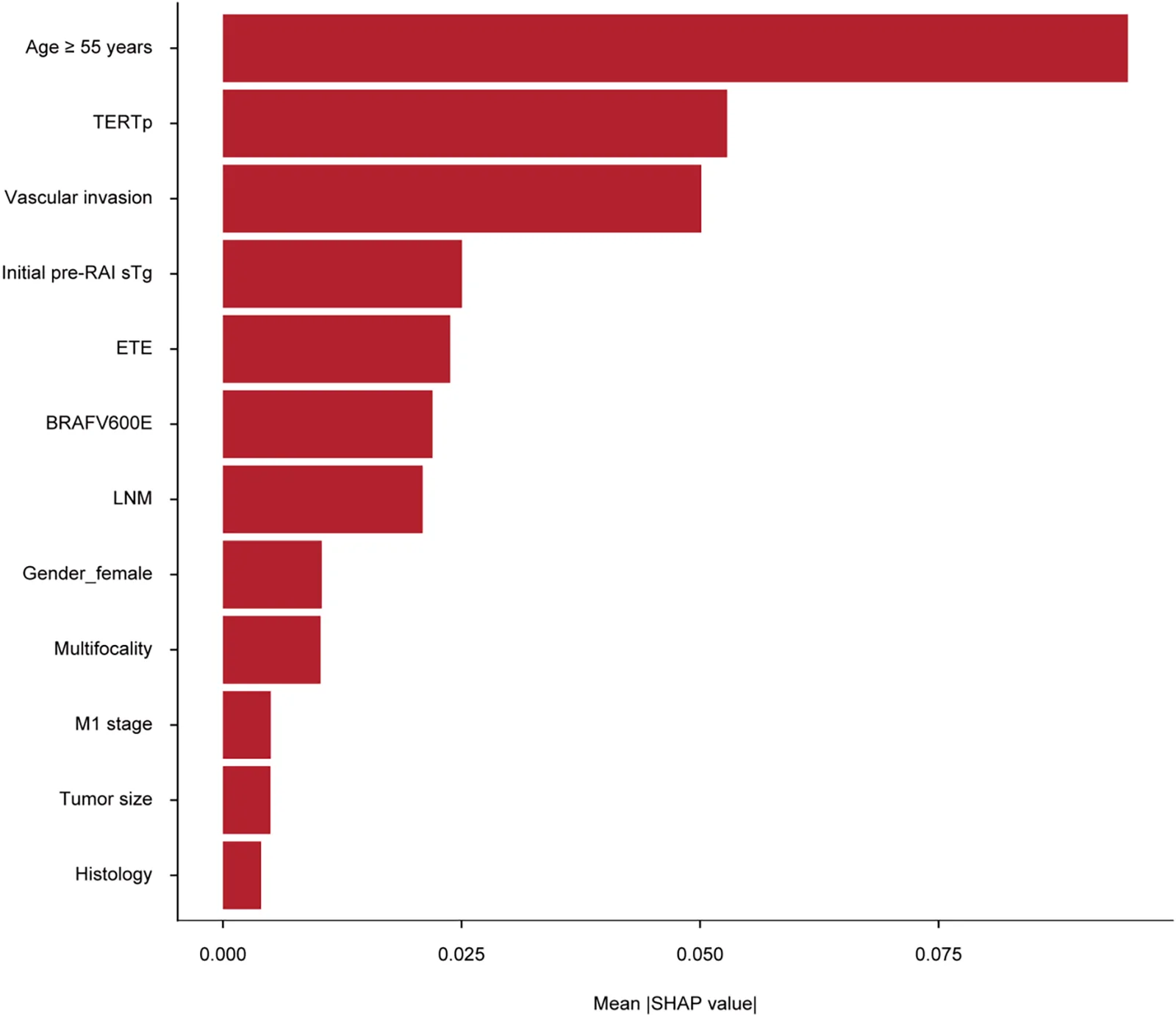
Global feature importance of the random-forest model. Mean absolute SHAP values rank the overall contribution of baseline predictors to RAIR-DTC predictions. Larger values indicate greater average influence on the model output.

**Figure 5:**
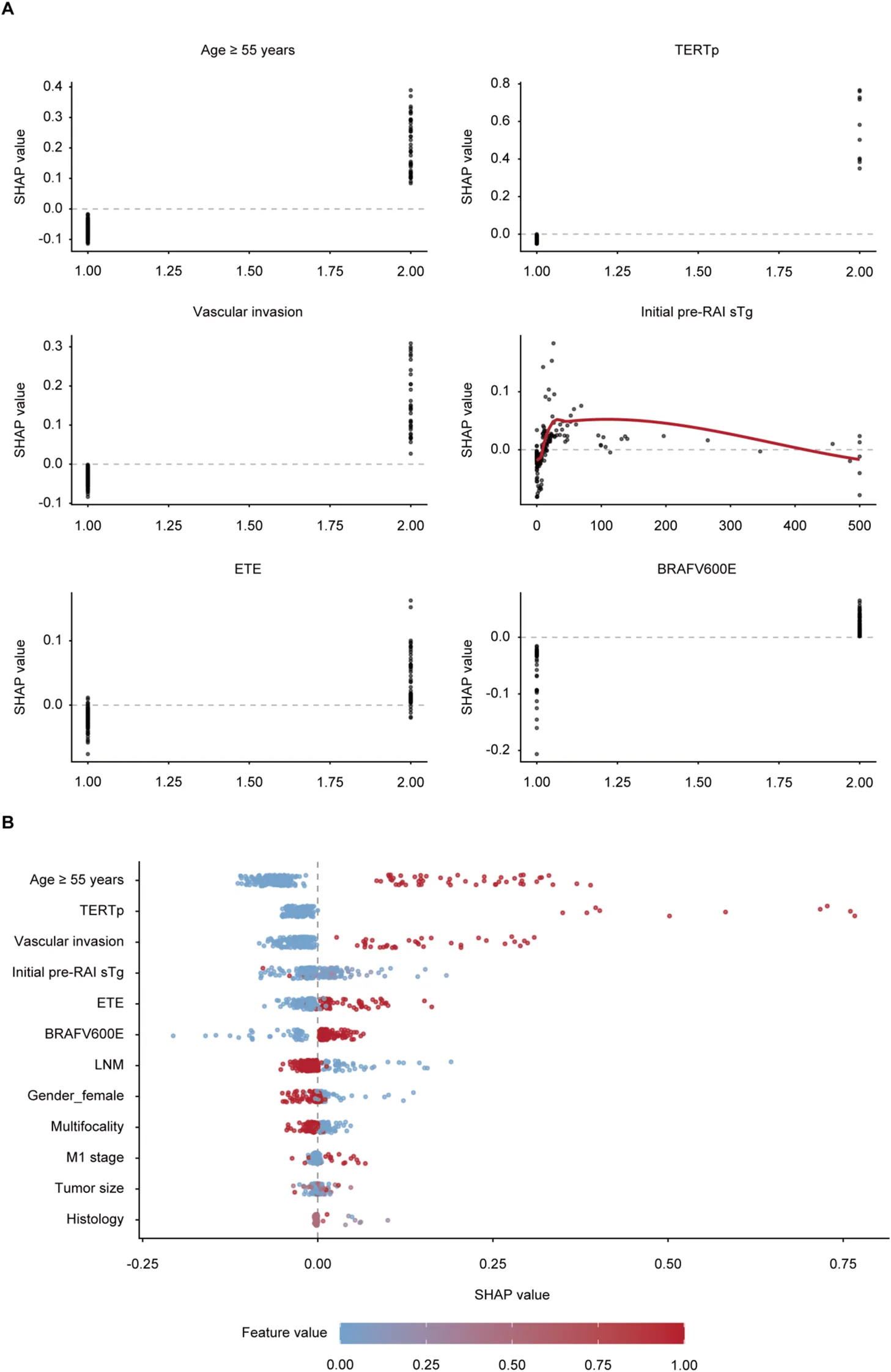
SHAP-based interpretation of the random-forest model. **(A)** SHAP dependence plots for the major predictors. **(B)** Beeswarm plot showing patient-level contributions. Positive SHAP values increase predicted RAIR-DTC risk and negative values decrease it. Color denotes the feature value.

### 3.5 Calibration and clinical utility

Calibration was evaluated using the raw predicted probabilities from the random forest model. In the internal test set, the calibration intercept was 0.843, the calibration slope was 0.366, and the observed-to-expected (O/E) ratio was 2.129, indicating that the model’s predicted risks were systematically lower than the observed event rate (mean predicted risk 0.073 vs. observed 0.156). In the external validation cohort, calibration deteriorated markedly, with an intercept of -0.841, a slope of 0.304, and an O/E ratio of 0.613 (mean predicted risk 0.241 vs. observed 0.148), demonstrating systematic overprediction of absolute risk in the new setting (**Supplementary Table S12**; **Figures 6A, B**). These findings highlight that preserved discrimination does not guarantee transportable probability estimates, and local recalibration would be necessary before absolute predicted probabilities are used for clinical decisions. Decision-curve analysis is presented as exploratory clinical-utility evidence rather than proof of clinical impact. Decision curve analysis suggested positive net benefit for several machine learning models over clinically relevant threshold ranges, although the advantage narrowed at higher thresholds (**Figures 7A, B**). Classification metrics across candidate decision thresholds further illustrated the trade-off between sensitivity and specificity in the internal and external cohorts (**Figures 8A, B**).

**Figure 6:**
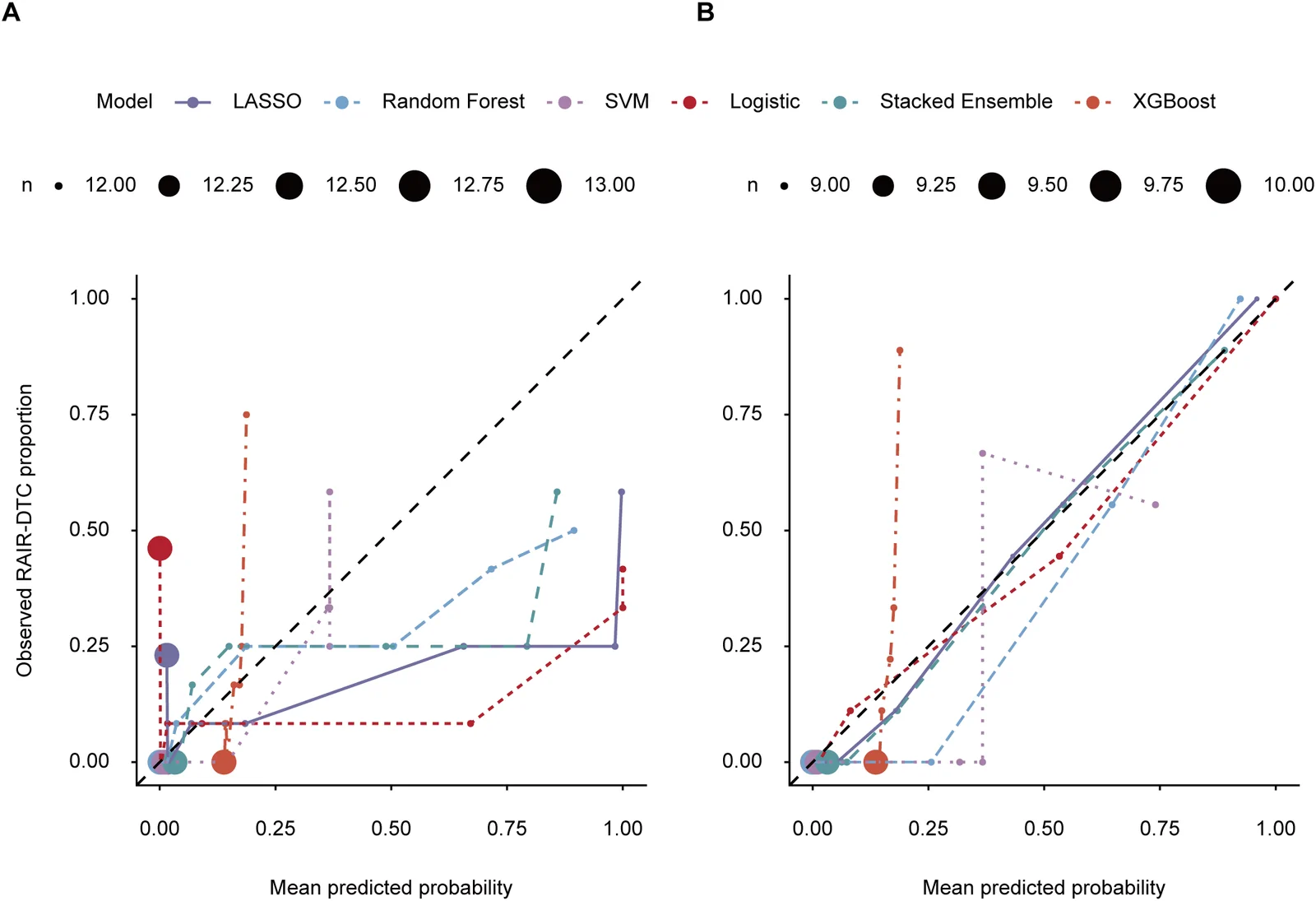
Model-specific calibration curves in the internal **(A)** and external **(B)** test cohort. Mean predicted probabilities are compared with observed RAIR-DTC proportions across grouped predictions; the diagonal line represents perfect calibration.

**Figure 7:**
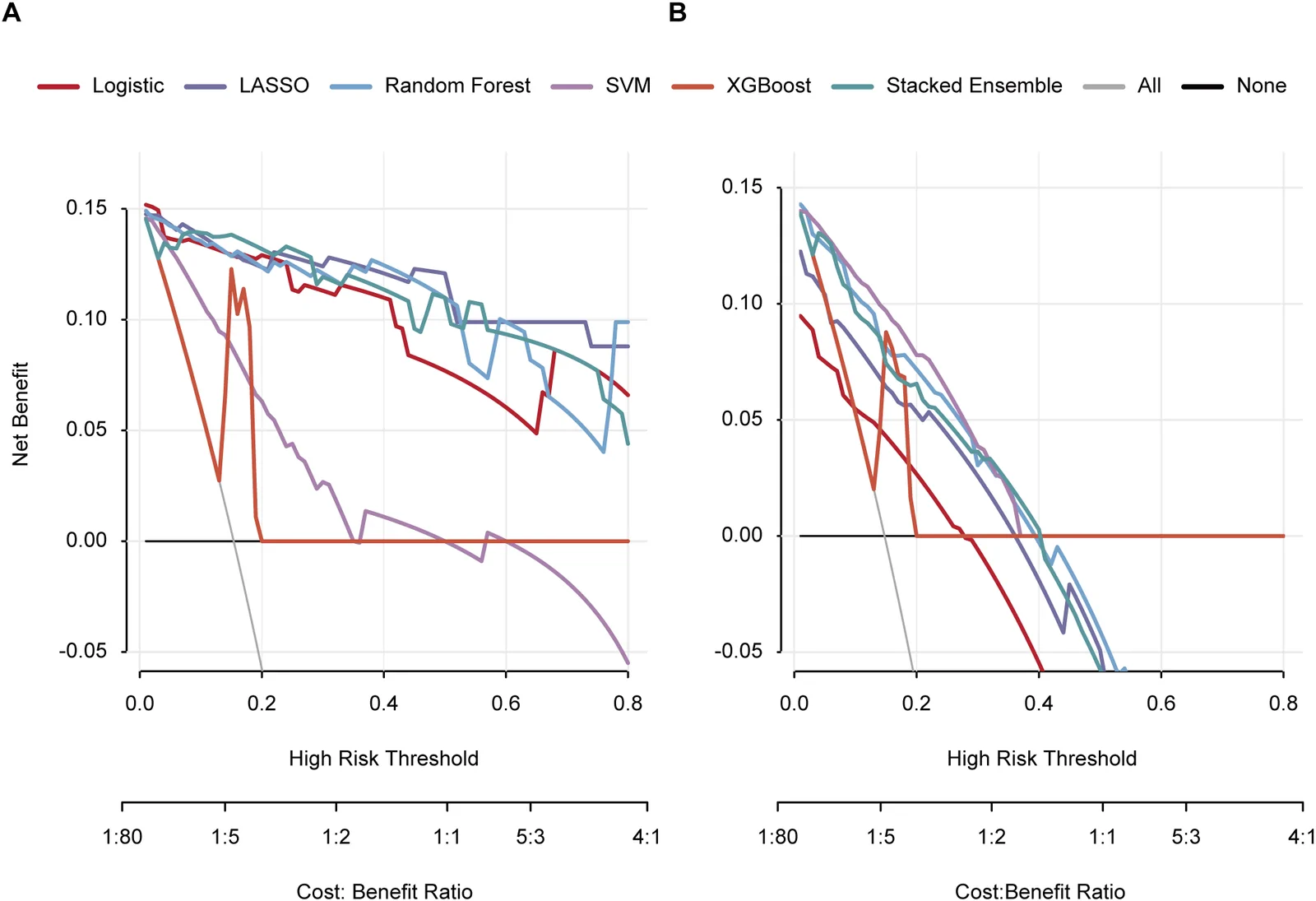
The panel presents net benefit across threshold probabilities in the internal **(A)** and external **(B)** test cohort, with treat-all and treat-none strategies as references. The decision curves quantify clinical net benefit across thresholds.

**Figure 8:**
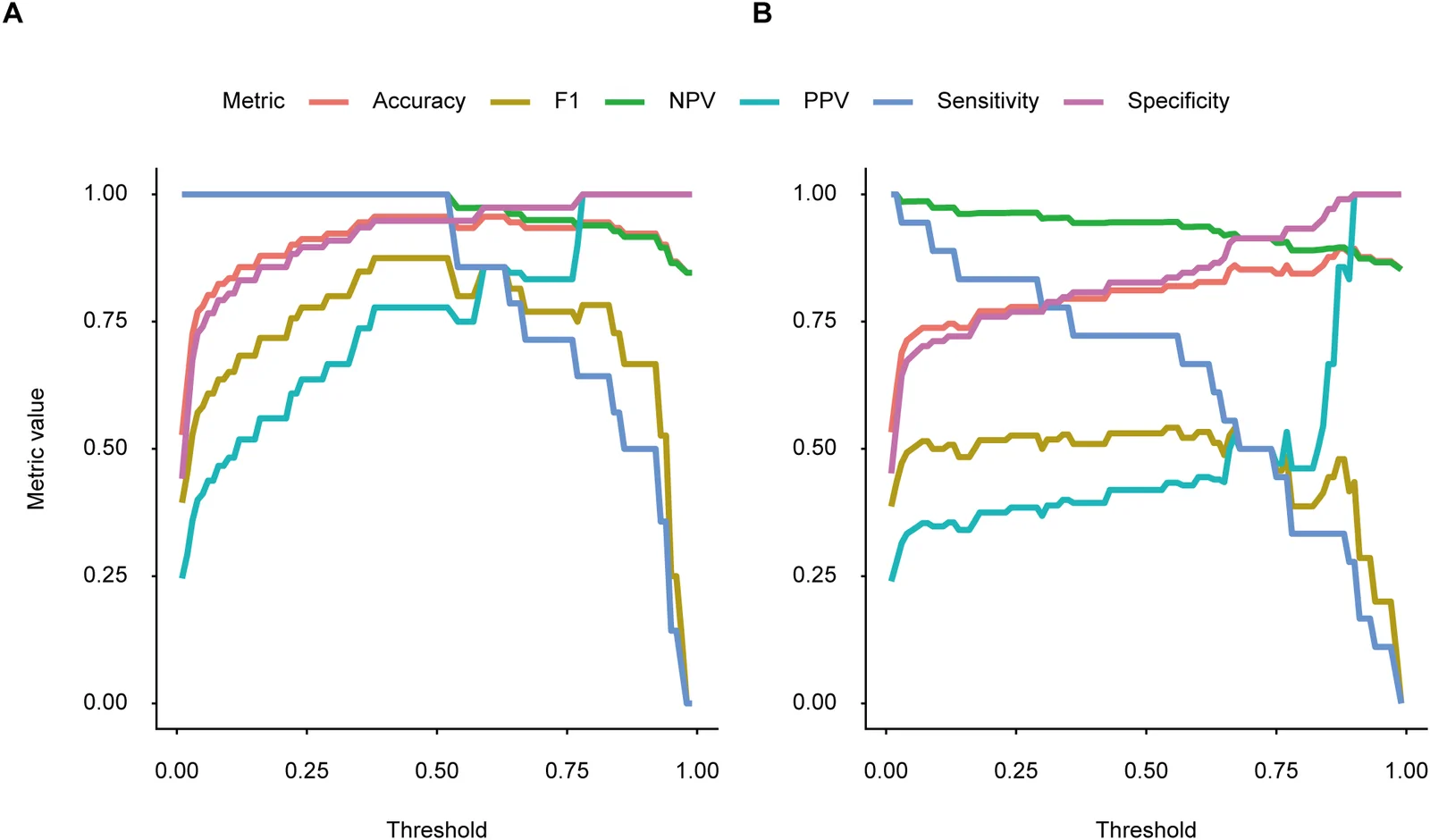
The panel displays accuracy, F1 score, NPV, PPV, sensitivity, and specificity across thresholds in the internal **(A)** and external **(B)** test cohort. Classification metrics are shown across candidate thresholds, illustrating the clinical trade-off between sensitivity and specificity.

### 3.6 Clinical risk stratification based on predicted probability thresholds

Using the fixed training-derived quantile cut points (33rd and 67th percentiles, corresponding to predicted risks of 0.000 and 0.003), the internal test set contained 61 low-risk, 13 intermediate-risk, and 16 high-risk patients. Two RAIR-DTC events occurred in the low-risk group (3.3%; 95% CI, 0.4%-11.3%), none in the intermediate-risk group (0.0%; 95% CI, 0.0%-24.7%), whereas 12 of 16 high-risk patients (75.0%; 95% CI, 47.6%-92.7%) developed RAIR-DTC. In the external cohort, the corresponding group sizes were 66, 13, and 43, with observed event rates of 0.0% (95% CI, 0.0%-5.4%), 15.4% (95% CI, 1.9%-45.4%), and 37.2% (95% CI, 23.0%-53.3%), respectively (**Table 5**).

**Table 5:** Random-forest risk groups and observed RAIR-DTC incidence using training-derived 33rd and 67th percentile cut points.

Cohort	Risk group	n	Mean predicted risk	Observed RAIR-DTC (95% CI)
Internal test	Low	61	0.000	2/61 (3.3%; 0.4%-11.3%)
	Intermediate	13	0.003	0/13 (0.0%; 0.0%-24.7%)
	High	16	0.408	12/16 (75.0%; 47.6%-92.7%)
External validation	Low	66	0.000	0/66 (0.0%; 0.0%-5.4%)
	Intermediate	13	0.003	2/13 (15.4%; 1.9%-45.4%)
	High	43	0.682	16/43 (37.2%; 23.0%-53.3%)

Risk groups were defined by the 33rd and 67th percentiles of the predicted-risk distribution in the training set (cutoffs: 0.000 and 0.003). 95% confidence intervals for binomial proportions were calculated using the Clopper-Pearson method.

### 3.7 Subgroup analyses

Subgroup discrimination was heterogeneous and several estimates were based on small samples. In the internal test set, AUC was 0.603 among patients aged <55 years and 0.824 among those aged ≥55 years; estimates were higher in *BRAF* V600E-mutant disease (0.905), TERTp-wild-type disease (0.879), M0 disease (0.863), and the very small M1 subgroup (1.000; n=9), while some strata were not estimable because they contained no events or all events. In the external cohort, AUCs remained favorable in patients aged <55 years (0.877), *BRAF* V600E-mutant disease (0.948), TERTp-wild-type disease (0.928), and M0 disease (0.875), but were lower in the BRAF-wild-type (0.344), TERTp-mutant (0.780), and M1 (0.683) subgroups. These exploratory analyses should not be interpreted as definitive evidence of subgroup-specific model failure or benefit (**Supplementary Table S13**).

## 4 Discussion

### 4.1 Major findings

The present study developed and externally validated an explainable, leakage-aware machine learning framework for the early prediction of RAIR-DTC using only variables available before or at the date of first postoperative RAI administration. Unlike many previously reported prediction models that incorporated post-treatment variables or treatment-response information, our model was deliberately designed to estimate future RAIR transformation at the earliest clinically actionable time point. Among six candidate algorithms, random forest achieved the most favorable balance between discrimination, probability accuracy, robustness, and transportability. External validation showed that although discrimination remained strong across institutions for all models (external AUCs ranging from 0.854 to 0.893, with random forest at 0.892), calibration deteriorated considerably, indicating that preservation of patient ranking does not necessarily translate into reliable absolute risk estimation. Herein, RAIR-DTC represents a heterogeneous composite clinical state encompassing primary radioiodine non-avidity, acquired loss of avidity, spatially heterogeneous uptake, and progression despite retained uptake. The current model predicts the composite endpoint and was not powered to develop criterion-specific models.

Beyond predictive performance, our study provides several clinically relevant contributions. First, by restricting predictors to baseline demographic, clinicopathological, molecular, and biochemical variables, the proposed model enables individualized risk estimation at the date of first postoperative RAI administration, before structural progression or radioiodine refractoriness becomes clinically evident. Second, SHAP-based explainability demonstrated that age, TERTp mutation, vascular invasion, sTg, and ETE consistently contributed most to model predictions, supporting the biological plausibility of the learned decision process (16). Third, the training-derived three-tier risk stratification identified a very-low-risk subgroup with zero events in the external validation cohort and a low event rate (3.3%) in the internal test set, while enriching the high-risk subgroup to event rates of 75.0% and 37.2%, respectively. Together, these findings suggest that explainable machine learning can support individualized surveillance strategies while simultaneously emphasizing that responsible clinical implementation requires rigorous external validation and local recalibration.

### 4.2 Clinical implications and potential implementation

The potential clinical value of the proposed model lies not merely in its predictive accuracy but in its ability to support individualized surveillance and earlier clinical decision-making. Because all predictors are routinely available before or at the date of first postoperative RAI administration, individualized risk estimates can be generated at the index date without requiring prolonged follow-up or evidence of structural progression. This timing is particularly important because opportunities for intervention may be greatest before complete loss of radioiodine avidity has occurred.

For patients classified as high risk, the model might help identify those who could benefit from closer surveillance, such as more frequent serum thyroglobulin monitoring (e.g., every 3–6 months during early follow-up) or earlier cross-sectional imaging when biochemical progression is suspected. However, these are hypotheses that require prospective evaluation before they can be translated into clinical recommendations.

Conversely, for patients predicted to be at low risk, the model suggests a very low event rate. In the external validation cohort, no RAIR-DTC events occurred within this group (0.0%; 95% CI, 0.0%-5.4%), although the internal test set showed a low but non-zero event rate of 3.3% (95% CI, 0.4%-11.3%). Therefore, while the low-risk category may support standard surveillance, the confidence intervals indicate that the true risk is not necessarily zero. Any suggestions regarding de-escalation of surveillance should be considered hypothesis-generating. Avoiding excessive imaging examinations, repeated PET/CT studies, or indiscriminate genomic testing may reduce healthcare costs, minimize patient anxiety, and improve allocation of clinical resources. The proposed model is not intended to replace physician judgment, imaging evaluation, or existing guideline recommendations. Instead, it should be regarded as an early-warning system that assists clinicians in tailoring surveillance intensity according to individualized baseline risk while allowing subsequent management decisions to remain guided by comprehensive clinical assessment and dynamic treatment response. These recommendations should be viewed as hypotheses for future prospective evaluation rather than as established clinical strategies derived from the current model.

### 4.3 Comparison with existing risk stratification systems

An important advantage of the present framework is that it complements rather than replaces the risk stratification systems currently recommended for DTC (2, 9, 10). Conventional systems, including the AJCC TNM staging system, the ATA recurrence risk classification, and dynamic risk stratification (DRS), have substantially improved prognostic assessment and postoperative management (2, 9, 10). However, these systems were primarily developed to estimate disease-specific mortality, recurrence risk, or treatment response rather than to predict future transformation into radioiodine-refractory disease. Consequently, they are not specifically designed to identify patients who are likely to lose iodine avidity before refractoriness becomes clinically apparent.

The TNM staging system focuses predominantly on long-term survival and incorporates variables such as age, tumor size, nodal involvement, and distant metastasis. Although these factors are associated with disease severity, they provide limited information regarding the biological mechanisms responsible for dedifferentiation and impaired iodine metabolism. Likewise, the ATA recurrence risk classification effectively stratifies postoperative recurrence risk but does not explicitly incorporate molecular alterations such as TERTp mutation or quantify complex interactions between clinicopathological and biochemical variables. Dynamic risk stratification further improves prognostic accuracy by integrating treatment response during follow-up; however, this approach inherently depends on post-treatment information, including serial thyroglobulin measurements and imaging findings, which are unavailable at the time when early prediction would be most clinically valuable.

In contrast, our machine learning framework addresses a distinct clinical question: estimating the probability of future RAIR transformation using only information available before or at the date of first postoperative RAI administration. This design allows clinicians to recognize patients at increased risk before irreversible dedifferentiation becomes clinically evident and before repeated ineffective RAI administration occurs. Integrating baseline machine learning prediction with conventional clinicopathological staging and subsequent dynamic response assessment may provide a more comprehensive strategy for longitudinal risk-adapted management than any individual system alone.

### 4.4 Machine learning performance and methodological implications

Among the six evaluated algorithms, random forest was selected for its overall balance rather than for the highest discrimination alone. Importantly, the final model selection was based entirely on out-of-fold results from the training set; the external validation cohort was not used for model selection, hyperparameter tuning, or any other aspect of model development, and external performance was assessed only after the final model was locked. Random forest had the lowest OOF Brier score (0.059) and an OOF AUC (0.944) within 0.001 of XGBoost (0.945). External AUCs were likewise nearly identical for random forest (0.892) and XGBoost (0.893). These results support random forest as a reasonable balance of discrimination and probability accuracy, but they do not establish statistical superiority over the other high-performing algorithms. Tree-based ensembles can flexibly model nonlinear relationships and interactions without requiring explicit specification of interaction terms; however, in this dataset, greater algorithmic complexity did not translate into a clear external discrimination advantage.

The external performance of conventional logistic regression (AUC 0.879) was close to that of several machine-learning models, suggesting that logistic regression can still provide useful discrimination when developed with the same leakage-aware feature set. Tumor dedifferentiation is plausibly driven by interacting molecular and clinicopathological factors whose effects may not follow a simple additive log-linear relationship. Ensemble tree-based approaches can accommodate threshold effects, interactions, and nonlinear risk patterns, but the stacked ensemble did not meaningfully improve on random forest in this dataset. This observation reinforces that increasing algorithmic complexity does not inevitably improve clinical prediction, particularly when base learners capture overlapping information or when event numbers are limited. Model selection should therefore emphasize reproducibility, calibration, and transportability rather than small within-sample differences in discrimination.

An equally important methodological contribution of our study is the explicit adoption of a leakage-aware modeling strategy. Information leakage remains a major concern in clinical machine learning, particularly in oncology, where post-treatment imaging findings, treatment response variables, cumulative treatment exposure, or follow-up outcomes are frequently incorporated into prediction models despite being unavailable at the intended prediction time. Such practices substantially inflate apparent model performance while limiting real-world applicability. By excluding all post-treatment, response-related, and time-dependent variables *a priori*, our framework more closely reflects the actual clinical workflow in which physicians must estimate future RAIR risk at the index date. Consequently, the predictive performance reported here likely represents a more realistic estimate of future deployment performance than many previously published models.

### 4.5 Biological interpretation of the predictive features

The biological interpretation of the model further supports its clinical credibility. SHAP analyses consistently identified older age, TERTp mutation, vascular invasion, elevated initial sTg, and ETE as the dominant determinants of predicted RAIR-DTC risk. These variables should not be interpreted as isolated predictors but rather as components of an interconnected biological network underlying thyroid cancer dedifferentiation. Their combined contribution likely reflects cumulative effects on tumor aggressiveness, metastatic potential, and progressive loss of iodine-handling capacity, processes that are inherently nonlinear and therefore particularly suitable for machine learning approaches.

Age remained the strongest contributor to model predictions. Although incorporated into contemporary staging systems primarily because of its prognostic value, increasing age also reflects broader biological changes associated with genomic instability, impaired immune surveillance, and accumulation of molecular alterations that favor aggressive tumor behavior. Likewise, TERTp mutation has emerged as one of the principal molecular drivers of thyroid cancer dedifferentiation. Experimental studies have demonstrated that TERT activation cooperates with MAPK pathway signaling, particularly in *BRAF* V600E-mutant tumors, to suppress NIS expression, impair iodine uptake, and promote cellular dedifferentiation (11). These mechanisms provide a plausible biological explanation for the prominent contribution of TERTp mutation observed in our model and are consistent with previous reports linking TERT alterations to radioiodine-refractory disease (12).

Vascular invasion and ETE similarly represent manifestations of invasive tumor biology rather than merely adverse pathological findings (13). Both features indicate increased metastatic capability and greater likelihood of residual microscopic disease, thereby reducing the effectiveness of radioiodine therapy. Initial sTg serves as an integrated biochemical surrogate of overall tumor burden, reflecting contributions from thyroid remnant tissue, LNM, and distant metastatic deposits (15, 17). SHAP dependence analysis suggested a nonlinear association between sTg and predicted RAIR risk, with risk increasing rapidly at lower concentrations before gradually plateauing at higher values. This pattern may reflect biological saturation, whereby extremely elevated sTg often characterizes patients with extensive disease who already possess advanced dedifferentiation or heterogeneous iodine avidity. Such nonlinear relationships are difficult to capture using conventional regression approaches but are naturally accommodated by tree-based machine learning algorithms. Consequently, rather than identifying entirely new biomarkers, the present model appears to uncover clinically meaningful interactions among established risk factors, thereby providing a more comprehensive representation of the biological processes leading to RAIR transformation. Because these contributions are made explicit through SHAP, the model may also improve clinician acceptance and support shared decision-making. However, it is important to emphasize that SHAP values describe the behavior of the fitted model and should not be overinterpreted as establishing causal effects or independent biological mechanisms. The model identifies predictive patterns, which are consistent with known biology, but does not provide mechanistic proof.

### 4.6 External calibration drift and implications for clinical deployment

A key methodological finding is that excellent discrimination did not guarantee reliable probability estimation after external validation. Although the random forest model maintained robust external discrimination with an AUC approaching 0.90, calibration deteriorated substantially, as reflected by the reduced calibration slope (0.304) and an observed-to-expected ratio of 0.613, indicating systematic overprediction of absolute risk in the external cohort. This discrepancy highlights a fundamental but frequently overlooked principle in clinical artificial intelligence: models that accurately rank patients according to relative risk may nevertheless provide poorly calibrated probability estimates when transported to new clinical environments.

Most published machine learning studies in oncology emphasize discrimination metrics such as ROC AUC while providing limited assessment of calibration or model transportability (18, 19). However, discrimination and calibration evaluate different aspects of model performance. A high AUC indicates that patients with events generally receive higher predicted risks than those without events, whereas calibration determines whether predicted probabilities correspond to actual event frequencies. Clinical decision-making often depends on absolute risk thresholds rather than relative ranking alone (18, 19). Consequently, poorly calibrated models may lead to inappropriate surveillance intensity, unnecessary investigations, or delayed intervention despite apparently excellent discrimination.

Several factors likely contributed to the calibration drift observed in the external cohort. Differences in molecular case mix, including substantially higher frequencies of *BRAF* V600E and TERTp mutations, may have shifted the baseline risk distribution between institutions. In addition, variations in thyroglobulin assay platforms, pathological interpretation of vascular invasion or ETE, RAI treatment protocols, imaging strategies, and follow-up intensity may all influence the relationships between predictors and outcomes. These findings illustrate the broader phenomenon of dataset shift that has increasingly been recognized as one of the major barriers to successful deployment of clinical artificial intelligence models across healthcare systems.

Calibration deterioration should not be interpreted as model failure but as a predictable and correctable phenomenon. Unlike discrimination, calibration can usually be restored through relatively simple recalibration procedures, such as updating the model intercept and slope using institution-specific data while preserving the underlying ranking function (20). Therefore, our findings support a practical deployment strategy in which external validation is routinely accompanied by local recalibration before probability-based clinical decisions are made. Whereas many previous prediction studies have concluded after demonstrating satisfactory AUC values, our results emphasize that successful clinical translation requires comprehensive evaluation of discrimination, calibration, and transportability. Such an implementation-oriented framework is likely to become increasingly important as machine learning models move from methodological development toward routine clinical practice.

### 4.7 Limitations

Several limitations should be acknowledged when interpreting our findings. First, the retrospective nature of this study inevitably introduces potential selection bias and information bias. Although consecutive eligible patients were included and outcome definitions followed contemporary clinical criteria, unmeasured factors such as variations in iodine exposure history, treatment adherence, comorbid conditions, or institutional management strategies may have influenced both predictor distributions and RAIR-DTC development. Prospective studies with standardized data collection protocols are therefore required to confirm the clinical effectiveness of the model.

Second, the number of RAIR-DTC events remained relatively limited, with 46 events in the development cohort and 18 events in the external validation cohort. Apparent training-set AUCs approached 0.98 for several models and should be interpreted cautiously because they reflect performance on data used for model fitting. The out-of-fold estimate for random forest (AUC 0.944) and the held-out internal test estimate (AUC 0.896) provide more realistic assessments of internal discrimination, although both remain imprecise because of the small event count. Repeated cross-validation and external testing were used to reduce overfitting, but the limited number of events constrained reliable characterization of rare interactions, extensive subgroup analyses, and highly complex models. In particular, subgroup findings involving TERTp mutation, distant metastasis, and BRAF-wild-type disease should be interpreted cautiously because small strata produce unstable estimates. Larger multicenter datasets with more events will be necessary to refine model complexity and evaluate performance across clinically relevant subgroups.

Third, RAIR-DTC represents a time-dependent clinical process rather than a simple static binary outcome. During follow-up, some patients may die from competing causes or develop other clinically relevant disease trajectories before RAIR transformation occurs. The current model does not explicitly account for competing risks or time-to-event information, which may limit individualized prediction of when RAIR-DTC is likely to develop. Future studies incorporating competing-risk frameworks or survival machine learning approaches may provide more accurate estimates of both event probability and timing.

Fourth, although the external validation cohort originated from a single additional institution within the same geographic region, broader generalizability across healthcare systems, ethnic populations, laboratory platforms, and RAI treatment strategies remains uncertain. Multicenter international validation with harmonized predictor definitions and standardized outcome adjudication will be essential before broad clinical deployment. Finally, although calibration deterioration was carefully evaluated, recalibration requires additional local data, and the optimal frequency and methodology for model updating in clinical practice remain to be established.

### 4.8 Future directions

Future research should focus on transforming this baseline prediction framework into a more comprehensive, dynamic, and globally applicable clinical decision-support system. First, because RAIR-DTC development is inherently longitudinal, future models should integrate time-to-event information using survival machine learning approaches, such as random survival forests, gradient boosting survival models, or deep survival networks. Such approaches may allow prediction not only of whether RAIR-DTC will occur but also when clinically meaningful risk escalation is expected, enabling more precise timing of surveillance and intervention. Because the primary outcome was binary and follow-up duration varied among patients, the resulting probabilities should not be interpreted as fixed 3-, 5-, or 10-year absolute risks. Dedicated time-to-event prediction models accounting explicitly for censoring will be required to estimate horizon-specific RAIR-DTC risk. Follow-up duration differed between patients with and without RAIR-DTC. Consequently, use of a binary outcome may result in differential outcome ascertainment and does not fully account for censoring. Formal assay harmonization across institutions should also be documented in future multicenter work.

Second, incorporation of additional biological and imaging biomarkers may further improve predictive performance. Circulating tumor DNA, DNA methylation patterns, microRNA signatures, and other liquid biopsy markers may provide dynamic indicators of tumor evolution and dedifferentiation. Meanwhile, radiomics features extracted from ultrasound, CT, or ¹^8^F-FDG PET/CT may capture imaging phenotypes associated with aggressive tumor biology that are not reflected by conventional radiological interpretation (21). Combining molecular, biochemical, and imaging signatures may ultimately provide a multidimensional representation of RAIR transformation (15).

Third, future multicenter collaborations should explore federated learning frameworks that enable model development across institutions without requiring direct sharing of patient-level data. Such approaches may overcome limitations caused by relatively rare RAIR-DTC events, improve population diversity, and facilitate continuous model updating while preserving patient privacy. Establishing standardized data definitions and collaborative validation networks will be particularly important for rare but clinically significant outcomes such as RAIR-DTC.

## 5 Conclusions

A leakage-aware random forest model integrating molecular, biochemical, and clinicopathological variables showed strong discrimination for RAIR-DTC and separated exploratory three-tier risk groups in internal and external cohorts. External discrimination was preserved despite meaningful case-mix shift, but calibration of the raw probabilities was poor in the external cohort. These findings support further prospective multicenter validation and site-specific recalibration before routine probability-based clinical use.

## Data Availability

The data generated in this study are publicly available in the NCBI BioProject database at http://www.ncbi.nlm.nih.gov/bioproject/1427906.
