# Supplementary material for: An explainable and leakage-aware machine learning model for early risk stratification of radioiodine-refractory differentiated thyroid cancer: development and single-center geographic external validation
Source: Front Endocrinol (Lausanne). 2026 Oct 2;17:1952443. doi: 10.3389/fendo.2026.1952443 (PMC13672023; doi:10.3389/fendo.2026.1952443)
Supplement: Supplementary file 1 [file Table1.docx]

**Supplementary Tables**

| **Supplementary Table S1. Distribution of RAIR-DTC diagnostic criteria among the 64 patients with RAIR‑DTC (development and external cohorts combined).** | | |
| --- | --- | --- |
| Criterion* | n | % of all RAIR‑DTC patients |
| 1. Non‑avid from initial post‑treatment 131I‑WBS | 37 | 57.81% |
| 2. Loss of previously documented avidity | 1 | 1.56% |
| 3. Heterogeneous uptake (avid and non‑avid lesions) | 8 | 12.50% |
| 4. Progression despite demonstrable uptake | 22 | 34.37% |
| 5. Cumulative RAI dose ≥600 mCi | 3 | 4.69% |
| *Patients could meet multiple criteria; percentages sum to >100%. Criteria definitions are provided in the Methods section. | | |

| **Supplementary Table S2. Clinical course in 64 patients from initial RAI to RAIR-DTC** | | |
| --- | --- | --- |
| Patients | Inclusion criteria | Detailed description of diagnostic basis |
| P1 | 1 | Rx-WBS indicates non-iodine uptake. The metastatic lesions do not exhibit iodine uptake in the Rx-WBS after the initial treatment, but the disease progresses. |
| P2 | 1 | Rx-WBS indicates non-iodine uptake. The metastatic lesions do not exhibit iodine uptake in the Rx-WBS after the initial treatment, but the disease progresses. |
| P3 | 3, 5 | The Rx-WBS showed positive uptake of iodine in the neck, but the ^18^F-FDG scan indicated positive metastases in the lungs and bones (not taking up iodine but detected), and the cumulative dose of ^131^I was 900 mCi > 600 mCi. |
| P4 | 4 | The Rx-WBS shows bilateral lung iodine uptake, with the Tg level rising from 48.1 to 1412.1, and the disease progression. |
| P5 | 4 | The Rx-WBS test was positive (indicating iodine uptake), but the Tg level increased from 52.3 to 601, and the disease progressed. |
| P6 | 1 | Rx-WBS indicates non-iodine uptake. The metastatic lesions do not exhibit iodine uptake in the Rx-WBS after the initial treatment, but the disease progresses. |
| P7 | 1 | Rx-WBS indicates non-iodine uptake. The metastatic lesions do not exhibit iodine uptake in the Rx-WBS after the initial treatment, but the disease progresses. |
| P8 | 1 | Rx-WBS indicates non-iodine uptake. The metastatic lesions do not exhibit iodine uptake in the Rx-WBS after the initial treatment, but the disease progresses. |
| P9 | 4 | The Rx-WBS shows multiple bone iodine uptake, and the Tg level increased from 201 to 528, indicating disease progression. |
| P10 | 1 | Rx-WBS indicates non-iodine uptake. The metastatic lesions do not exhibit iodine uptake in the Rx-WBS after the initial treatment, but the disease progresses. |
| P11 | 1 | Rx-WBS indicates non-iodine uptake. The metastatic lesions do not exhibit iodine uptake in the Rx-WBS after the initial treatment, but the disease progresses. |
| P12 | 1 | The Rx-WBS test showed no iodine uptake, but the Tg level increased from 9.4 to 755.4, and the disease progressed. |
| P13 | 1 | Rx-WBS indicates non-iodine uptake. The metastatic lesions do not exhibit iodine uptake in the Rx-WBS after the initial treatment, but the disease progresses. |
| P14 | 1 | Rx-WBS indicates non-iodine uptake. The metastatic lesions do not exhibit iodine uptake in the Rx-WBS after the initial treatment, but the disease progresses. |
| P15 | 3 | The Rx-WBS shows iodine uptake in the cervical lymph nodes, but no iodine uptake in the lungs, and the ^18^F-FDG showed positive results in both the neck and the lungs. |
| P16 | 1 | Rx-WBS indicates non-iodine uptake. The metastatic lesions do not exhibit iodine uptake in the Rx-WBS after the initial treatment, but the disease progresses. |
| P17 | 3 | The Rx-WBS showed iodine uptake in the clavicular region and parasternal lymph nodes, while the ^18^F-FDG revealed positive results in bilateral lungs. |
| P18 | 3 | The Rx-WBS showed iodine uptake in the multiple bones, but not in the bilateral lungs. |
| P19 | 1 | Rx-WBS indicates non-iodine uptake. The metastatic lesions do not exhibit iodine uptake in the Rx-WBS after the initial treatment, but the disease progresses. |
| P20 | 2 | The Rx-WBS shows radioactive iodine uptake, but the period from the initial treatment to the diagnosis of RAIR-DTC was very long (from August 1992 to February 2023), suggesting a gradual loss of iodine uptake ability. |
| P21 | 1 | Rx-WBS indicates non-iodine uptake. The metastatic lesions do not exhibit iodine uptake in the Rx-WBS after the initial treatment, but the disease progresses. |
| P22 | 1 | Rx-WBS indicates non-iodine uptake. The metastatic lesions do not exhibit iodine uptake in the Rx-WBS after the initial treatment, but the disease progresses. |
| P23 | 1 | Rx-WBS indicates non-iodine uptake. The metastatic lesions do not exhibit iodine uptake in the Rx-WBS after the initial treatment, but the disease progresses. |
| P24 | 1 | Rx-WBS indicates non-iodine uptake. The metastatic lesions do not exhibit iodine uptake in the Rx-WBS after the initial treatment, but the disease progresses. |
| P25 | 4 | The Rx-WBS test shows positive for bone (iodine uptake) and an extremely high Tg level (74,568), indicating disease progression. |
| P26 | 1 | The Rx-WBS test showed no iodine uptake, but the ^18^F-FDG test was positive, and the Tg level increased from 464 to 4464, indicating disease progression. |
| P27 | 1 | The Rx-WBS showed no iodine uptake, but the Tg level increased from 20.7 to 705.4, and the patient was dead. |
| P28 | 1 | Rx-WBS indicates non-iodine uptake. The metastatic lesions do not exhibit iodine uptake in the Rx-WBS after the initial treatment, but the disease progresses. |
| P29 | 4 | The Rx-WBS shows radioactive iodine uptake in the bilateral lungs and bones,and the Tg level increased from 46.6 to 704, indicating disease progression. |
| P30 | 1 | Rx-WBS indicates non-iodine uptake. The metastatic lesions do not exhibit iodine uptake in the Rx-WBS after the initial treatment, but the disease progresses. |
| P31 | 4 | Rx-WBS showed radioactive iodine uptake. Tg level increased from 500 to 2595, and the disease progressed. |
| P32 | 1, 4 | The Rx-WBS was negative, and the sTg level increased from 26.7 to 289. The survival status was death, suggesting disease progression. |
| P33 | 1 | Rx-WBS indicates non-iodine uptake. The metastatic lesions do not exhibit iodine uptake in the Rx-WBS after the initial treatment, but the disease progresses. |
| P34 | 4 | The Rx-WBS shows iodine uptake in bilateral lungs and bones, and the Tg level increased from 57.7 to 1038, reflecting the progression of the disease. |
| P35 | 4 | The Rx-WBS showed iodine uptake in the thoracic vertebrae and the spinal cord, and the Tg level increased from 12.1 to 4997, indicating disease progression. |
| P36 | 1 | Rx-WBS indicates non-iodine uptake. The metastatic lesions do not exhibit iodine uptake in the Rx-WBS after the initial treatment, but the disease progresses. |
| P37 | 1 | Rx-WBS indicates non-iodine uptake. The metastatic lesions do not exhibit iodine uptake in the Rx-WBS after the initial treatment, but the disease progresses. |
| P38 | 1 | Rx-WBS indicates non-iodine uptake. The metastatic lesions do not exhibit iodine uptake in the Rx-WBS after the initial treatment, but the disease progresses. |
| P39 | 1 | Rx-WBS indicates non-iodine uptake. The metastatic lesions do not exhibit iodine uptake in the Rx-WBS after the initial treatment, but the disease progresses. |
| P40 | 3, 4 | The Rx-WBS showed radioactive iodine uptake in the neck, and ^18^F-FDG showed positive in the lungs and bones. The Tg level increased from 47.4 to 743.3, suggesting disease progression. |
| P41 | 1 | Rx-WBS indicates non-iodine uptake. The metastatic lesions do not exhibit iodine uptake in the Rx-WBS after the initial treatment, but the disease progresses. |
| P42 | 1 | Rx-WBS indicates non-iodine uptake. The metastatic lesions do not exhibit iodine uptake in the Rx-WBS after the initial treatment, but the disease progresses. |
| P43 | 3 | The Rx-WBS showed radioactive iodine uptake in the subclavian and mediastinal lymph nodes, and ^18^F-FDG showed positive. The Tg level increased from 15.1 to 399.2, suggesting disease progression. |
| P44 | 3, 4 | The Rx-WBS showed radioactive iodine uptake in the bilateral cervical lymph nodes, and 18F-FDG showed positive in the bilateral lungs and bones. The Tg level increased from 4.8 to 425.0, suggesting disease progression. |
| P45 | 1 | Rx-WBS indicates non-iodine uptake. The metastatic lesions do not exhibit iodine uptake in the Rx-WBS after the initial treatment, but the disease progresses. |
| P46 | 1 | Rx-WBS indicates non-iodine uptake. The metastatic lesions do not exhibit iodine uptake in the Rx-WBS after the initial treatment, but the disease progresses. |
| P47 | 3, 4 | The Rx-WBS showed radioactive iodine uptake in the right supraclavicular and hilar lymph nodes, and ^18^F-FDG showed positive in the bilateral lungs and sternum. The Tg level increased from 58.8 to 848.4, suggesting disease progression. |
| P48 | 1 | Rx-WBS indicates non-iodine uptake. The metastatic lesions do not exhibit iodine uptake in the Rx-WBS after the initial treatment, but the disease progresses. |
| P49 | 1 | Rx-WBS indicates non-iodine uptake. The metastatic lesions do not exhibit iodine uptake in the Rx-WBS after the initial treatment, but the disease progresses. |
| P50 | 1 | Rx-WBS indicates non-iodine uptake. The metastatic lesions do not exhibit iodine uptake in the Rx-WBS after the initial treatment, but the disease progresses. |
| P51 | 1 | The Rx-WBS test showed no iodine uptake, although the Tg level was less than 0.04 because Tg is suppressed by TSH. |
| P52 | 1 | The Rx-WBS shows no iodine uptake, but the ^18^F-FDG indicates positive results for hilar lymph nodes and multiple bones. |
| P53 | 4 | The Rx-WBS showed iodine uptake in the neck, clavicle, mediastinal lymph nodes, and the Tg level ranged from 6.9 to 458.1, indicating disease progression. |
| P54 | 4 | The Rx-WBS showed radioactive iodine uptake in the bilateral lungs and multiple bones. The Tg level increased from 11.2 to 864.4. |
| P55 | 4, 5 | The Rx-WBS showed radioactive iodine uptake in the multiple bones. The Tg level increased from 16.0 to 875.9, and the time from the initial treatment to the diagnosis of RAIR-DTC was short (from September 2013 to May 2022), suggesting disease progression. Moreover, the cumulative dose reached 1100 mCi. |
| P56 | 4 | The Rx-WBS shows iodine uptake in the cervical lymph nodes, lungs and bones, |
| P57 | 1 | Rx-WBS indicates non-iodine uptake. The metastatic lesions do not exhibit iodine uptake in the Rx-WBS after the initial treatment, but the disease progresses. |
| P58 | 1 | Rx-WBS indicates non-iodine uptake. The metastatic lesions do not exhibit iodine uptake in the Rx-WBS after the initial treatment, but the disease progresses. |
| P59 | 4 | The Rx-WBS shows iodine uptake in the bilateral lungs and bone, and the Tg level increased from 1.2 to 633.8, indicating disease progression. |
| P60 | 4 | Rx-WBS showed radioactive iodine uptake. Tg level increased from 0.5 to 900.3, and the patient was dead. |
| P61 | 4, 5 | The Rx-WBS showed radioactive iodine uptake in the multiple bones. The Tg level increased from 62.31 to 438.5, and the time from the initial treatment to the diagnosis of RAIR-DTC was short (from September 2019 to December 2019). Moreover, the cumulative dose reached 600 mCi. |
| P62 | 4 | The Rx-WBS shows iodine uptake in bilateral lungs, and the Tg level increased from 60.0 to 811.0, reflecting the progression of the disease. |
| P63 | 4 | The Rx-WBS shows iodine uptake in multiple bones, and the Tg level increased from 0.3 to 302.6, reflecting the progression of the disease. |
| P64 | 4 | The Rx-WBS shows bilateral lung and multiple bone iodine uptake, and the Tg level increased from 39.0 to 189.9. |

| **Supplementary Table S3. Timing of predictor measurements relative to the index date (first RAI administration).** | |
| --- | --- |
| Predictor | Measurement time/window |
| Age | At diagnosis (retrieved from medical records) |
| Sex | At diagnosis |
| Histology | Post‑surgical pathology |
| Tumor size | Post‑surgical pathology (maximum diameter) |
| Multifocality | Post‑surgical pathology |
| ETE | Post‑surgical pathology |
| Vascular invasion | Post‑surgical pathology |
| LNM | Post‑surgical pathology (central/lateral) |
| Distant metastasis | Pre‑RAI imaging (CT, PET/CT, etc.) |
| BRAFV600E | Post‑surgical tumor tissue (NGS) |
| TERTp mutation | Post‑surgical tumor tissue (NGS) |
| sTg (stimulated Tg) | After thyroid hormone withdrawal (3–4 weeks; TSH >30 mIU/L), immediately before the date of first postoperative RAI administration |
| All predictors were collected at or before the index date. | |

| **Supplementary Table S4. Exact coding of variables used in machine learning models.** | | |
| --- | --- | --- |
| Variable | Type | Coding |
| Age | Binary | 0 = <55 years, 1 = ≥55 years |
| Sex | Binary | 0 = Male, 1 = Female |
| Histology | Binary | 0 = PTC, 1 = FTC (other variants grouped as PTC) |
| Tumor size | Continuous | Maximum tumor diameter (cm) |
| Multifocality | Binary | 0 = Singleness, 1 = Multifocality (≥2 foci) |
| ETE | Binary | 0 = Absent, 1 = Present |
| Vascular invasion | Binary | 0 = Absent, 1 = Present |
| LNM | Binary | 0 = Absent, 1 = Present (any N1) |
| Distant metastasis | Binary | 0 = M0, 1 = M1 |
| BRAFV600E | Binary | 0 = Wild‑type, 1 = Mutant |
| TERTp mutation | Binary | 0 = Wild‑type, 1 = Mutant (C228T or C250T) |
| sTg | Continuous | Stimulated thyroglobulin (ng/mL) before first RAI |

| **Supplementary Table S5. Tuned hyperparameters and search ranges of candidate models.** | | | |
| --- | --- | --- | --- |
| Model | Parameter | Search Range/Grid | Final Value |
| Logistic | None | NA | NA |
| LASSO | Penalty(lambda) | 0.001-0.1 (by 0.001) | 0.014 |
| RandomForest | mtry | 2-10 (integer) | 6 |
| RandomForest | min.node.size | 1-5 (integer) | 3 |
| RandomForest | ntree | Fixed at 1000 | 1000 |
| RandomForest | classwt | c (0.5,0.5) | c(0.5, 0.5) |
| SVM | Cost | 0.01-10 (log scale) | 0.225 |
| SVM | RBF sigma | 0.1-5 (log scale) | 1.334 |
| SVM | Margin | 0.01-0.5 | 0.081 |
| XGBoost | nrounds(trees) | 100-1000 (by 50) | 507 |
| XGBoost | max_depth | 1-6 (integer) | 1 |
| XGBoost | min_child_weight | 1-5 (integer) | 3 |
| XGBoost | eta(learning rate) | 0.01-0.3 (by 0.01) | 0.01 |
| XGBoost | gamma(loss reduction) | 0-0.5 (by 0.001) | 0.001 |
| XGBoost | subsample | 0.5-1.0 (by 0.05) | 0.672 |
| Stacked Ensemble | meta-learner | Logistic (default) | Logistic (default) |
| **Note:** The hyperparameter search space for each algorithm is provided in the "Search Range" column. For Random Forest, class weights were set to equal (0.5,0.5) to treat both classes equally during the tree-building process. For XGBoost, early stopping was applied with a patience of 50 rounds, resulting in an optimal boosting iteration of 507 rounds. | | | |

| **Supplementary Table S6. Missing data frequencies per variable (overall <2%).** | | |
| --- | --- | --- |
| Variable | Missing n | Missing % |
| Age | 0 | 0 |
| Sex | 0 | 0 |
| Histology | 0 | 0 |
| Tumor size | 2 | 0.5 |
| Multifocality | 0 | 0 |
| ETE | 1 | 0.2 |
| Vascular invasion | 2 | 0.5 |
| LNM | 1 | 0.2 |
| Distant metastasis | 0 | 0 |
| BRAFV600E | 0 | 0 |
| TERTp mutation | 0 | 0 |
| sTg | 3 | 0.7 |
| Imputation was performed within cross‑validation folds using median (continuous) or mode (categorical) from the training fold. | | |

| **Supplementary Table S7. Training-derived Youden thresholds for binary classification.** | |
| --- | --- |
| Model | Cut-off |
| Logistic | 0.12486 |
| LASSO | 0.161124 |
| RandomForest | 0.245 |
| SVM | 0.075318 |
| XGBoost | 0.120727 |
| StackedEnsemble | 0.037547 |

| **Supplementary Table S8. Exploratory survival analysis (Cox proportional hazards model) for time to RAIR-DTC development in the development cohort.** | | |
| --- | --- | --- |
| Variable | HR (95% CI) | P value |
| Age ≥ 55 years | 3.21(1.89-5.45) | <0.001 |
| TERTp mutation | 2.98(1.76-5.04) | <0.001 |
| Vascular invasion | 2.12(1.28-3.51) | 0.003 |
| sTg (per 10‑unit increase) | 1.08(1.02-1.14) | 0.012 |
| ETE | 1.78(0.98-3.24) | 0.058 |
| BRAFV600E | 1.45(0.82-2.56) | 0.197 |
| LNM | 1.33(0.71-2.49) | 0.374 |
| Multifocality | 1.12(0.64-1.96) | 0.685 |
| Model was adjusted for all variables listed. HR = hazard ratio; CI = confidence interval. | | |

| **Supplementary Table S9. Distribution of RAIR-DTC diagnostic criteria among the 64 patients with RAIR-DTC.** | | |
| --- | --- | --- |
| Criterion* | **Development cohort (% of 64 RAIR‑DTC patients)**  n=46 (71.9) | **External validation cohort (% of 64 RAIR‑DTC patients)**  n=18 (28.1) |
| 1. Metastases that are RAI-nonavid on the initial posttreatment 131I-WBS. | 27 (42.2) | 10 (15.6) |
| 2. Functional metastases that originally are RAI-avid gradually lose their iodine uptake ability. | 1 (1.6) | 0 (0.0) |
| 3. Some metastases are RAI-avid, while others are RAI-nonavid, which can be detected by other imaging methods such as 18F-FDG PET/CT, CT, or MRI. | 6 (9.4) | 2 (3.1) |
| 4. RAI-avid metastases maintain the iodine uptake ability, but the disease progresses within one year after RAI therapy, including gradual growth of the lesions, the appearance of new lesions, or a continuous increase of Tg or Tg-antibody (TgAb) levels. | 16 (25.0) | 6 (9.4) |
| 5. Cumulative RAI dose ≥600 mCi. | 2 (3.1) | 1 (1.6) |
| *Patients could meet multiple criteria; percentages sum to >100%. Criteria definitions are provided in the Methods section. | | |

| **Supplementary Table S10. Standardized mean differences (SMD) for case-mix shift between internal and external cohorts.** | |
| --- | --- |
| Variable | SMD (Internal vs External) |
| RAIR‑DTC event | 0.022 |
| Age ≥55 years | 0.301 |
| sTg >10.77 ng/mL | 0.053 |
| Tumor size ≥2 cm | 0.105 |
| Distant metastasis | 0.121 |
| ETE | 0.045 |
| Vascular invasion | 0.015 |
| LNM | 0.167 |
| Multifocality | 0.182 |
| BRAFV600E mutation | 0.446 |
| TERTp mutation | 0.676 |
| SMD > 0.2 indicates a potentially meaningful difference. | |

| **Supplementary Table S11. Comparison of discrimination between the random forest model and a simplified ATA high-risk indicator.** | | |
| --- | --- | --- |
| Cohort | Model | AUC (95% CI) |
| Internal test | Random Forest | 0.896 (0.781-1.000) |
|  | Simplified ATA high-risk indicator* | 0.651 (0.505-0.794) |
| External validation | Random Forest | 0.892 (0.829-0.955) |
|  | Simplified ATA high-risk indicator* | 0.749 (0.629-0.846) |
| *Operational definition: high risk = T4 disease and/or distant metastasis (M1) present at baseline; all remaining patients were grouped as intermediate/low risk. The rule was fixed independently of RAIR-DTC outcome and was not tuned to the validation data. | | |

| **Supplementary Table S12. Random-forest calibration statistics based on raw prediction probabilities.** | | | | | |
| --- | --- | --- | --- | --- | --- |
| Cohort | Intercept | Slope | O/E ratio | Mean predicted risk | Observed event rate |
| Internal test | 0.843 | 0.366 | 2.129 | 0.073 | 0.156 |
| External validation | -0.841 | 0.304 | 0.613 | 0.241 | 0.148 |
| O/E = observed event rate / mean predicted risk. Calibration statistics were calculated using raw predicted probabilities. | | | | | |

| **Supplementary Table S13. Exploratory subgroup AUC analysis for the current random forest model with 95% confidence intervals.** | | | | |
| --- | --- | --- | --- | --- |
| Cohort | Subgroup | Level | N | AUC (95% CI) |
| Internal test | Age | <55 | 71 | 0.603 (0.373-1.000) |
|  |  | ≥55 | 19 | 0.824 (0.589-0.993) |
|  | BRAFV600E | Mutant | 66 | 0.905 (0.782-0.999) |
|  |  | WT | 24 | Not estimable (no events) |
|  | TERTp | WT | 88 | 0.879 (0.737-0.992) |
|  |  | Mutant | 2 | Not estimable (all events) |
|  | M stage | M0 | 81 | 0.863 (0.693-0.992) |
|  |  | M1 | 9 | 1.000 (1.000-1.000) |
| External validation | Age | <55 | 80 | 0.877 (0.799-0.954) |
|  |  | ≥55 | 42 | 0.764 (0.594-0.899) |
|  | BRAFV600E | Mutant | 110 | 0.948 (0.899-0.985) |
|  |  | WT | 12 | 0.344 (0.000-0.667) |
|  | TERTp | WT | 93 | 0.928 (0.851-0.985) |
|  |  | Mutant | 29 | 0.780 (0.586-0.958) |
|  | M stage | M0 | 105 | 0.875 (0.768-0.971) |
|  |  | M1 | 17 | 0.683 (0.416-0.924) |
| All subgroup analyses are exploratory and should be interpreted cautiously because of small sample sizes and low event counts in several strata. CIs were estimated using 2,000 patient-level bootstrap resamples. | | | | |

**Supplementary methods**

**Postoperative RAI and serologic testing**

All patients in this study underwent thyroid hormone withdrawal (levothyroxine sodium) for 3-4 weeks combined with a low-iodine diet until TSH >30 mU/L. An appropriate RAI dose (3.7-7.4 GBq) was administered based on clinical indications. Serum TgAb, sTg, and TSH were measured using electrochemiluminescence immunoassay (Roche E170, Switzerland) or chemiluminescent immunoassay (ADVIA Centaur, Bayer, Germany), according to the laboratory platform available during the study period. The reported analytical measurement range for sTg was 0.04-1000.00 ng/mL (reference range, 1.40-78.00 ng/mL), with an analytical sensitivity of 0.04 ng/mL. Values below or above the measurement range were recorded as 0.04 ng/mL or 1000.00 ng/mL, respectively, for statistical analysis. TgAb was assessed concurrently; the reference range was 0-115 kU/L, and values <115 kU/L were considered negative. To minimize interference from anti-thyroglobulin antibodies, six patients with elevated TgAb were excluded from the sTg based analyses. The TSH reference range was 0.27-4.20 mU/L. All patients received levothyroxine TSH suppression therapy during and after RAI treatment, with a target TSH level <0.1 mU/L. The sTg level at the first RAI and at RAIR-DTC diagnosis were used as dynamic serological indicators. Because two immunoassay platforms were used over the retrospective study period and no formal cross-platform calibration study was performed, residual inter-assay variability cannot be excluded; this issue was considered when interpreting absolute sTg thresholds.

**Imaging diagnosis**

Patients were evaluated using neck ultrasound, chest CT, ¹⁸F-FDG PET/CT, bone scintigraphy, ¹³¹I-WBS, serial changes in sTg and thyroglobulin antibody (TgAb) levels from the initial RAI to RAIR-DTC diagnosis, and assessment of lymph-node and distant metastases. Stimulated serologic assessments were performed after thyroid hormone withdrawal with TSH >30 mIU/L and without known exogenous iodine interference. Post-therapeutic ¹³¹I-WBS was acquired at the protocol-defined time point 4 days after RAI administration. In contrast, because of the retrospective real‑world design, ¹⁸F-FDG PET/CT and other cross‑sectional imaging were clinically indicated and were not obtained at fixed calendar intervals for every patient. The longitudinal RAIR-DTC classification therefore used all available serial examinations and required concordant structural/imaging evidence for progression when Criterion 4 was applied. For dual-modal phenotyping, a ¹³¹I-WBS(+)/FDG(+) ("dual-avid" or dual-positive) phenotype was defined at the patient-assessment level as the presence of at least one pathological radioiodine-avid lesion and at least one pathological FDG-avid lesion during the corresponding dual-modal evaluation; the two signals were not required to arise from the same anatomical lesion. Thus, spatially discordant lesions could contribute to the dual-positive phenotype.

**¹³¹I whole-body scan (¹³¹I-WBS) and SPECT/CT**

**Instrumentation and reagents**

Imaging was performed using a Siemens Symbia T6 SPECT/CT scanner (Siemens Healthineers, Germany) equipped with a high‑energy general‑purpose parallel‑hole collimator. The imaging agent was an oral solution of ¹³¹I with a radionuclidic purity greater than 99% (Chengdu Zhonghe Gaotong Isotope Co., Ltd., China).

**Imaging protocol**

Patients underwent ¹³¹I-WBS and regional SPECT/CT fusion imaging 4 days after ¹³¹I therapy. For ¹³¹I-WBS, patients were positioned supine and scanning was performed at a speed of 18 cm/min using a 256 × 1024 matrix; the energy peak was set at 364 keV with a 20% window, and anterior and posterior projection images were acquired simultaneously. Based on the WBS findings, SPECT/CT tomographic imaging was then performed for regions of interest such as the neck, thorax, or any suspected foci of abnormal radiotracer uptake. SPECT acquisition used a high‑energy general‑purpose parallel‑hole collimator with the energy window centered at 364 keV ± 10%, a 128 × 128 matrix, an acquisition time of approximately 30 seconds per frame, and automatic body contour tracking. CT acquisition was performed with the integrated low‑dose spiral CT (tube voltage 120 kV, tube current 80 mA, slice thickness 3.75 mm) and used for anatomical localization and attenuation correction.

**Image processing**

Image reconstruction and fusion were performed on the manufacturer's dedicated workstation. SPECT images were iteratively reconstructed using the ordered-subsets expectation maximization (OSEM) algorithm with CT-based attenuation correction; coronal, sagittal, axial, and three-dimensional fusion images were generated using multi-planar reconstruction.

**Image analysis**

Images were reviewed independently by two experienced nuclear medicine physicians. Reviewers evaluated the imaging datasets independently of one another; discrepancies were resolved by consensus and, when necessary, adjudication by a senior nuclear medicine physician. Because this was a retrospective study, formal blinding to all longitudinal clinical information and genotype was not prospectively implemented, and this is acknowledged as a potential source of interpretation bias. Analysis focused on (i) localization of radioactive foci suggestive of residual thyroid tissue in the anterior neck region and (ii) identification of abnormal uptake suggestive of metastasis in cervical or mediastinal lymph nodes, lungs, bones, or other sites. Qualitative interpretation correlated radiotracer uptake with anatomical CT findings (e.g., lesion size, morphology, calcification, and bone destruction); physiological and nonspecific uptake were distinguished from pathological uptake.

**¹⁸F-FDG PET/CT imaging**

**Instrumentation and imaging protocol**

Imaging was performed using a UNITED IMAGING uMI 780 digital PET/CT system (United Imaging Healthcare, Shanghai, China), with data processed on a uWI-MI workstation. Patients fasted for at least 6 hours prior to the scan, with blood glucose controlled at ≤ 11.1 mmol/L. ¹⁸F-FDG (radiochemical purity > 95%; Jiangyuan ANTECO Pharmaceuticals, Nanjing, China) was intravenously injected at a dose of 3.70-5.55 MBq/kg. After resting for approximately 60 minutes post‑injection and voiding, patients underwent whole-body PET/CT scanning from the skull vertex to the mid-femur in list mode. CT parameters were as follows: tube voltage 120 kV, tube current 100 mA, slice thickness 3 mm, pitch 0.9875. PET acquisition used 3D mode with 4-5 bed positions, 2 minutes per bed position, and a 30% bed overlap.

**Image analysis**

PET images were reconstructed using the OSEM algorithm (2 iterations, 20 subsets) with time‑of‑flight, point‑spread function, and scatter and random corrections. The image matrix was 256 × 256 with 3 mm slice thickness and a 3 mm full‑width‑at‑half‑maximum Gaussian filter. CT images were used for attenuation correction and anatomical localization. All fused images were transferred to the uWI-MI workstation for visual and semi-quantitative analysis. Two nuclear medicine physicians with more than 5 years of experience independently reviewed axial, coronal, and sagittal images. A three-dimensional volume of interest was placed at the site of maximum metabolic activity to obtain SUVmax automatically. Background uptake was measured using a 3 cm spherical region of interest in normal right-lobe liver parenchyma. Discrepancies between readers were resolved by a third senior physician. The dual-positive phenotype was assigned at the patient-assessment level as defined above and did not imply that iodine and FDG uptake were co‑localized within an identical lesion.

**Molecular testing**

**Nucleic acid extraction**

Gene testing was performed on postoperative tumor tissue samples. Total nucleic acid (DNA and RNA) was extracted from formalin-fixed, paraffin-embedded tissue specimens using the QIAamp Mini Kit. DNA and RNA concentrations were quantified using a NanoDrop 1000 spectrophotometer.

**Library preparation and molecular panel**

A custom molecular panel was used, targeting single nucleotide variants (SNVs) in 11 genes (BRAF, HRAS, KRAS, NRAS, TP53, and TERT promoter [C228T and C250T]) and fusion variants in 5 genes (RET, NTRK1, NTRK3, PAX8, and THADA). Quality control requirements included a total DNA input greater than 10 ng per sample with intact DNA fragment integrity; DNA base Q30 and alignment rates greater than 80% and 90%, respectively; average sequencing depth greater than 500×; RNA target base Q30 rate greater than 80%; and total reads greater than 300,000. Sequencing libraries were prepared using the TruSeq DNA Library Preparation Kit.

**Next-generation sequencing (NGS) and variant calling**

DNA sequencing was performed on an Illumina NextSeq system using paired‑end reads (2×150 bp). Reads were aligned to the human reference genome GRCh37 using the Burrows-Wheeler Aligner (BWA). MuTect2 and GATK were used to identify SNVs and small insertions/deletions, respectively. All final candidate variants were visually verified using the Integrative Genomics Viewer (IGV).
